# Abnormal dynamic functional connectivity and topological properties of cerebellar network in male obstructive sleep apnea

**DOI:** 10.1111/cns.14786

**Published:** 2024-06-03

**Authors:** Lifeng Li, Ting Long, Yuting Liu, Muhammad Ayoub, Yucheng Song, Yongqiang Shu, Xiang Liu, Li Zeng, Ling Huang, Yumeng Liu, Yingke Deng, Haijun Li, Dechang Peng

**Affiliations:** ^1^ Department of Radiology, The First Affiliated Hospital, Jiangxi Medical College Nanchang University Nanchang Jiangxi Province China; ^2^ Department of Radiology, The Affiliated Changsha Central Hospital, Hengyang Medical School University of South China Hengyang Hunan Province China; ^3^ Department of Ophthalmology Hunan Children's Hospital Changsha Hunan Province China; ^4^ School of Computer Science and Engineering, Central South University Changsha Hunan Province China; ^5^ PET Center, The First Affiliated Hospital, Jiangxi Medical College Nanchang University Nanchang Jiangxi Province China

**Keywords:** cerebellum, dynamic functional connectivity, graph theory analysis, hidden Markov model, obstructive sleep apnea

## Abstract

**Purpose:**

To investigate dynamic functional connectivity (dFC) within the cerebellar‐whole brain network and dynamic topological properties of the cerebellar network in obstructive sleep apnea (OSA) patients.

**Methods:**

Sixty male patients and 60 male healthy controls were included. The sliding window method examined the fluctuations in cerebellum‐whole brain dFC and connection strength in OSA. Furthermore, graph theory metrics evaluated the dynamic topological properties of the cerebellar network. Additionally, hidden Markov modeling validated the robustness of the dFC. The correlations between the abovementioned measures and clinical assessments were assessed.

**Results:**

Two dynamic network states were characterized. State 2 exhibited a heightened frequency, longer fractional occupancy, and greater mean dwell time in OSA. The cerebellar networks and cerebrocerebellar dFC alterations were mainly located in the default mode network, frontoparietal network, somatomotor network, right cerebellar CrusI/II, and other networks. Global properties indicated aberrant cerebellar topology in OSA. Dynamic properties were correlated with clinical indicators primarily on emotion, cognition, and sleep.

**Conclusion:**

Abnormal dFC in male OSA may indicate an imbalance between the integration and segregation of brain networks, concurrent with global topological alterations. Abnormal default mode network interactions with high‐order and low‐level cognitive networks, disrupting their coordination, may impair the regulation of cognitive, emotional, and sleep functions in OSA.

## Graphical abstract

Utilizing dynamic functional connectivity and graph analyses, along with HMM validation, this study examines cerebellar network changes in OSA patients. Our findings demonstrated aberrant cerebellar FC and disrupted topology in OSA, along with maintaining function through coordination and compensation. Provides a framework for elucidating OSA neural mechanisms.

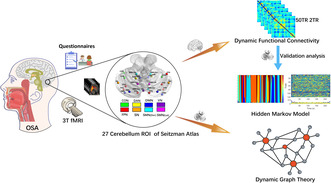

## 1 INTRODUCTION

Obstructive sleep apnea (OSA) is a sleep‐related breathing disorder characterized by frequent upper airway collapse during sleep, resulting in intermittent hypoxemia and sleep fragmentation. The prevalence of OSA ranges from 6% to 19% in adult women and up to 13% to 33% in adult men.^1^ Studies have demonstrated OSA can cause neurocognitive dysfunction, mood disorders, and daytime sleepiness, exhibiting disruptions in functional brain networks and alterations in topological properties.^2^, ^3^ OSA may impair axonal projections in the cerebellum, eliciting a state of hypoxia and oxidative stress in the cerebellum and leading to secondary neurological tissue compensation and interruption of connectivity pathways between the brain and cerebellum.^4^ Moreover, OSA‐induced sleep disturbances disrupt cerebellar neural reflexes and chronotropic regulation in areas controlling blood pressure and respiratory function, increasing the likelihood of cognitive dysfunction.^5^

The cerebellum is a structurally and functionally susceptible region in OSA patients, suggesting its potential involvement in the underlying mechanisms of OSA.^6^, ^7^, ^8^ Disruptions in functional connectivity (FC) within the cerebellum and other regions among OSA patients may serve as the foundation for compromised autonomic, cognitive, and sensory‐motor functions.^4^ In patients with OSA, the cerebellar network exhibits altered small‐world characteristics, along with changes in function and information transfer. Specifically, there is a cerebellar convexity in the network characterized by lower local efficiency and reduced clustering coefficients.^4^, ^9^ These findings affirm the occurrence of an imbalance between differentiation and integration in the cerebellar network of OSA patients, which is believed to be the mechanism underlying cognitive deficits in this population.^4^ Besides, it is crucial to examine the association between the cerebellum and cerebrum. OSA patients primarily experience changes in cerebrocerebellar functional connectivity due to structural modifications in the brain and impaired perfusion. This impact is specifically associated with sleep fragmentation and hypoxia, which are implicated in OSA‐related cognitive decline.^4^ Moreover, chronic intermittent hypoxia induces both functional and structural alterations in the connectivity between the cerebellum and the entire brain, potentially contributing to a spectrum of changes in mood, cognition, emotion, and sleep in individuals with OSA.^6^, ^10^ Therefore, we further analyzed the cerebrocerebellar dynamic functional connectivity (dFC) to determine the temporal variation in these characteristics.

Traditional static functional connectivity (sFC) overlooked the impact of time on network dynamics and the potential for functional processes to vary temporally.^11^ While dFC could capture more temporal variations in connectivity patterns over shorter timescales, and contribute to discriminating the OSA patients.^12^ Thus, elucidating the time‐varying integration and reconfiguration of brain networks is crucial for understanding intrinsic dynamics and responses to stimuli.^13^ The dFC is associated with cognitive impairment and clinical manifestations in OSA patients and may be a critical mechanism of neurocognitive dysfunction.^3^, ^14^ Prior studies have employed the hidden Markov model (HMM) to analyze dynamic transitions between cognitive brain states. This modeling approach could uncover hidden state features of brain networks.^15^ Therefore, we further leveraged HMM to validate the reliability of dFC patterns and mitigate potential biases. Additionally, graph theory can facilitate the examination of neuropathological mechanisms linked to cognitive disorders.^15^, ^16^ Analyzing dynamic graph theory (dGT) metrics of cerebellar networks using the sliding window (SW) method could provide key insights into the cerebellum's time‐varying topological organization.^17^

Based on the above findings, we hypothesized that the dFC of the cerebellar‐whole brain network and the dynamic topological properties of the cerebellar network would be affected in OSA patients. To test this, we first investigated alterations in cerebellar dFC and used the HMM to validate the robustness of the dFC results. The cerebellar topological properties and cerebrocerebellar dFC matrices were then examined via the SW approach. Finally, the relationships between temporal properties, topology, and clinical characteristics were analyzed to reveal potential cerebellar neural mechanisms in OSA.

## 2 MATERIALS AND METHODS

### 2.1 Participants

Sixty male patients and 60 male health controls (HC) were selected. The diagnostic criteria of OSA followed the 2007 guidelines from the American Academy of Sleep Medicine.^18^ The inclusion criteria were as follows: (1) aged 18 to 60 years male adults (Due to observed sex differences in the association between OSA and cognitive impairment, as well as variations in the developmental sequences and topological network structures of the cerebellum across different sexes, we opted to exclusively include male participants to mitigate potential gender‐related confounding effects)^19^, ^20^; (2) right‐handed; (3) apnea–hypopnea index (AHI) ≥15 events/h. The HC group was selected based on an AHI below 5 events/h. Exclusion criteria for both groups were: (1) history of hypertension, cardiovascular disease, or diabetes; (2) central nervous system disorders including epilepsy, psychosis, neurodegenerative diseases, prior head injuries, and depression; (3) structural brain lesions evident on MRI; (4) MRI contraindications; and (5) previous substance or psychotropic drug abuse.

### 2.2 Polysomnography

All participants underwent ≥7 h of comprehensive overnight polysomnography (PSG) monitoring and avoided caffeine, alcohol, and sleep medications the day before the sleep monitoring. Physiological parameters were recorded from 10 pm to 6 am the following morning using a Respironics LE‐Series system (Alice 5 LE, USA). Measured parameters included electrocardiogram, electroencephalogram (EEG), electromyography, electrooculogram, thoracic and abdominal respiratory movements, airflow, snoring, and body position. Obstructive apnea was defined according to standard criteria by a reduction in airflow ≥90% lasting ≥10 s (apnea); hypopnea was characterized as a reduction in the airflow by 30% lasting for ≥10 s accompanied by a reduction in oxygen saturation (SaO_2_) ≥ 3% and/or EEG arousal.^21^ The AHI was computed according to the average number of apnea and hypopnea events per hour during sleep. A diagnosis of OSA was made when AHI was ≥5 events/h. PSG calculates and reports the following: sleep stages (N1‐3), arousal index (AI), AHI, oxygen desaturation index (ODI), total sleep time, percentage of total sleep time spent at oxygen saturation <90% (SaO_2_ < 90%), nadir/mean SaO_2_, rapid eye movement (REM), sleep efficiency (SE), and other indicators.

### 2.3 Clinical and neuropsychological measures

Each participant completed a standardized series of neuropsychological assessments before scanning. The Epworth Sleepiness Scale (ESS) evaluates daytime sleepiness. The Pittsburgh Sleep Quality Index (PSQI) assessed subjective sleep quality and disturbances over the past month. The Montreal Cognitive Assessment (MoCA) was administered to assess cognitive function across eight domains.^22^ The Hamilton Anxiety Scale (HAMA) and Hamilton Depression Scale (HAMD) evaluated anxiety and depressive symptoms. The detailed process is described in Appendix S1: Method 1.

### 2.4 Resting‐state fMRI data acquisition

Magnetic resonance data were obtained using a Magnetom Trio 3.0 T MRI (Siemens, Munich, Germany) system scanner. The resting‐state functional magnetic resonance imaging (fMRI) data were acquired from a gradient echo planar imaging (EPI) sequence using the following parameters: echo time (TE) = 30 ms, repetition time (TR) = 2000 ms, thickness/gap = 4.0/1.2 mm, field of view (FOV) = 230 mm × 230 mm, flip angle = 90°, slices = 30, matrix = 64 × 64, duration = 8 min. Finally, high‐resolution 3D T1‐weighted brain structure MRI images were obtained using a magnetized fast gradient echo sequence with the following parameters: TE = 2.26 ms, TR = 1900 ms, thickness/gap = 1.0/0.5 mm, FOV = 250 mm × 250 mm, flip angle = 9°, matrix = 256 × 256, slices = 176.

### 2.5 Resting‐state fMRI data preprocessing

The fMRI data were preprocessed using the Data Processing and Analysis Brain Imaging (DPABI, https://rfmri.org/DPABI) software and the Statistical Parameter Mapping (SPM12, https://www.fil.ion.ucl.ac.uk/spm/software/spm12/) software, which was run on Matlab2018b. The procedures involved: (1) Discard the first 10 time points. The data would be eliminated if a participant's maximal head movement was more than 1.5 mm in any direction or if the angle rotation along any axis exceeded 1.5° across the time series. Ultimately, eight OSA patients and 7 HC were excluded. (2)Slice timing and three‐dimensional head motion correction; (3) Co‐register the T1‐weighted structural image; (4) Use the new segmentation method to segment the transformed structural image; (5) Normalize the functional data space to the Montreal Neurological Institute (MNI) template; (6) Regress out nuisance covariates; and (7) Use time band‐pass filtering. The detailed process was listed in Appendix S1: Method 2.

### 2.6 Dynamic functional network connectivity analysis

The Seitzman atlas divides the entire brain into 273 cerebral and 27 cerebellar regions, with the cerebellum further subdivided into 8 networks (Table S1, Figure S1).^16^ The average time series across 27 nodes was extracted with a radius of 4 mm as the region of interest (ROI). Dynamic FC metrics were computed with a SW of 50 TR and a step size of 2 TR in the DynamicBC toolkit (dynamic brain connectome, http://restfmri.net/forum/DynamicBC),^23^ according to prior literature.^24^, ^25^ Ninety‐one SW were then acquired. A K‐means clustering method and elbow method discerned optimal cluster numbers.^26^ Finally, the following time‐varying parameters were computed^27^: mean dwell time of dFC (MDTD), frequency (FR), and number of transitions (NT).

Furthermore, cerebrocerebellar dFC was computed between 27 cerebellar nodes and the whole brain, utilizing these 27 cerebellar nodes with a 4 mm radius as ROI. Alterations in cerebrocerebellar dFC were subsequently ascertained. For each voxel, the standard deviation of transformed dFC across windows was calculated and utilized as a measure of time‐varying connectivity.^28^

### 2.7 Dynamic graph theory analysis of dynamic functional networks

A dynamic graph theoretic approach was utilized to examine temporal fluctuations in the topological properties of functionally connected networks. All network analyses were performed with the Graph‐Theoretic Network Analysis toolkit (GRETNA, http://www.nitrc.org/projects/gretna) to investigate global and nodal topological attributes. The area under the curve was calculated for each network metric across a sparsity threshold ranging from 0.05 to 0.40 with an interval of 0.01.^29^ Furthermore, global and node properties were determined alongside the computation of detailed graph metrics. Detailed graph features are presented in Appendix S1: Method 3. Subsequently, the temporal variability of the graphical metrics was calculated.^30^

### 2.8 Verification analysis of network measures

HMM assumes time series data can be depicted by a finite set of state sequences.^31^ The number of states in HMM needs to be determined in advance.^32^ We computed feasible state values by referring to states obtained with alternative window widths and step sizes (30TR, 1TR and 30TR, 2TR) and previous studies to determine the optimal state quantity.^33^ The HMM‐multivariate autoregressive model (HMM‐MAR, https://github.com/OHBA‐analysis/HMM‐MAR) toolbox was applied to compute the HMM parameters. Three key metrics characterize the temporal dynamics of HMM states: fractional occupancy (FO), mean dwell time of HMM (MDTH), and switching rate (SR).^34^, ^35^, ^36^

### 2.9 Network‐based statistical analysis

To identify altered cerebellar subnetworks in OSA, we performed network‐based statistical analysis (NBS) between states 1 and 2. This nonparametric technique detects interconnected subcomponents within a broader network. Two‐sample *t*‐tests compared FC between states, yielding *t*‐statistics and *p*‐values for each edge. We thresholded edges at *p* = 0.0001 to construct the initial subnetwork. Permutation testing (5000 iterations) determined subnetwork significance against an empirical null distribution (*p* < 0.005), with age, years of education, body mass index (BMI), and head motion as covariates.^37^

### 2.10 Statistical analyses

Referring to previous literature,^38^ all skewness and kurtosis values computed to assess the normality of the demographic, neuropsychological, and clinical data were within the acceptable range (−2 and +2) in IBM SPSS 24.0 software. The two‐sample *t*‐test was used to analyze the normalized data; otherwise, the Mann‐Whitney nonparametric tests were used for non‐normalized data. The GRETNA toolbox's two‐sample *t*‐tests assessed between‐group network metric differences. Covariates included age, education, head motion, and BMI. We compared dFC, dynamic topological properties, and HMM metrics between two groups (*p* < 0.05). Node differences were false discovery rate (FDR)‐corrected at *p* < 0.05. Correction for multilevel comparison of cerebrocerebellar dFC results was performed using Gaussian random field theory (GRF) correction with a two‐tailed combined threshold of voxel‐level *p* < 0.005 and cluster‐level *p* < 0.05. Time‐varying features of dFC within the cerebellum, cerebrocerebellar connectivity abnormalities, HMM indices, and positive dGT indices in OSA, covarying for head motion, age, education, and BMI, were assessed using Spearman's correlation with neuropsychological and clinical scale scores. The threshold for statistical significance was set at *p* < 0.05.

## 3 RESULTS

### 3.1 Demographic and clinical characteristics data of the participants

Sixty OSA patients and sixty HC patients were included in this study. There were no significant differences in age, total sleep time, N2, years of education, and head movement between the two groups (*p* > 0.05). Additional clinical and demographic data is provided in Table 1.

**TABLE 1:** Demographic and clinical data between OSA patients and HC.

Characteristic	OSA patients (*N* = 60)	HC (*N* = 60)	*t*‐value	*p*‐value
Age, years	37.88 ± 9.08	41.25 ± 10.47	−1.88	0.062
BMI, kg/m^2^	26.90 ± 3.65	20.86 ± 1.63	11.68	<0.001*
AHI, events/h	49.42 ± 18.11	2.20 ± 1.18	20.16	<0.001*
Nadir SaO_2_, %	69.62 ± 11.94	93.77 ± 3.90	−14.90	<0.001*
Mean SaO_2_, %	92.53 ± 4.24	96.92 ± 2.25	−7.07	<0.001*
SaO_2_ < 90%	20.50 ± 17.58	0.78 ± 1.45	8.66	<0.001*
Total sleep time, min	390.63 ± 97.70	408.84 ± 24.50	−1.40	0.166
Sleep efficiency, %	0.86 ± 0.17	0.92 ± 0.04	−2.88	0.005*
N1, %	27.31 ± 16.44	10.26 ± 3.34	7.87	<0.001*
N2, %	38.69 ± 12.96	41.79 ± 6.25	−1.67	0.099
N3, %	20.44 ± 15.58	29.65 ± 5.26	−4.34	<0.001*
REM, %	13.57 ± 11.01	18.31 ± 5.70	−2.96	0.004*
AI, events/h	29.15 ± 18.04	11.62 ± 2.81	7.44	<0.001*
ODI, events/h	43.43 ± 22.89	1.73 ± 1.08	14.10	<0.001*
Education, years	12.07 ± 3.73	11.53 ± 3.17	0.84	0.400
ESS, scores	10.53 ± 4.36	1.52 ± 1.42	15.24	<0.001*
MoCA scale, scores	24.35 ± 2.89	28.00 ± 1.46	−8.72	<0.001*
PSQI, scores	7.88 ± 3.38	3.63 ± 1.65	8.76	<0.001*
HAMA, scores	8.98 ± 4.76	5.77 ± 2.76	4.53	<0.001*
HAMD, scores	8.47 ± 6.21	4.03 ± 2.77	5.05	<0.001*
Head movement, mm	0.13 ± 0.06	0.12 ± 0.06	1.43	0.155

*Note*: *p < 0.05, which was considered statistically significant, data are presented as the mean ± SD.

Abbreviations: AHI, apnea‐hypopnea index; AI, arousal index; BMI, body mass index; ESS, Epworth Sleepiness Scale; ESS, Epworth Sleepiness Scale; HAMA, Hamilton Anxiety Scale; HAMD, Hamilton Depression Scale; HC, healthy controls; MoCA, Montreal Cognitive Assessment; N, number; N/A, not applicable; ODI, oxygen desaturation index; OSA, obstructive sleep apnea; PSQI, Pittsburgh Sleep Quality Index; REM, rapid eye movement; SaO_2_ < 90%, percentage of total sleep time spent at oxygen saturation less than 90%; SaO_2_, oxygen saturation.

### 3.2 Dynamic functional connectivity time‐varying features

**FIGURE 1:**
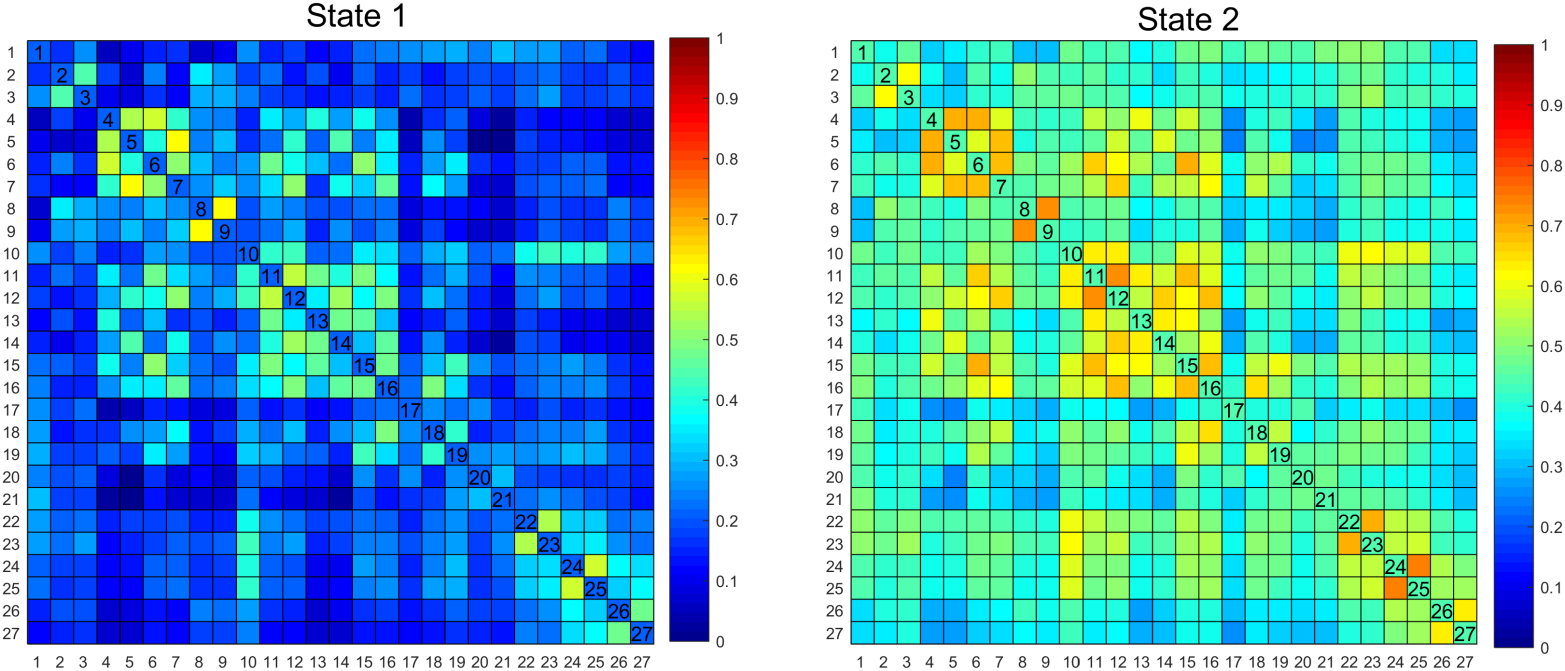
Cluster centroids of dynamic functional connectivity for all subjects in OSA patients and HC with a sliding window length of 50 TR and steps of 2 TR. HC, healthy controls; OSA, obstructive sleep apnea; TR, repetition time. The values ranging from 1 to 27 in the X and Y coordinates of the matrix, respectively, correspond to the eight distinct networks enumerated in Table 1.

**FIGURE 2:**
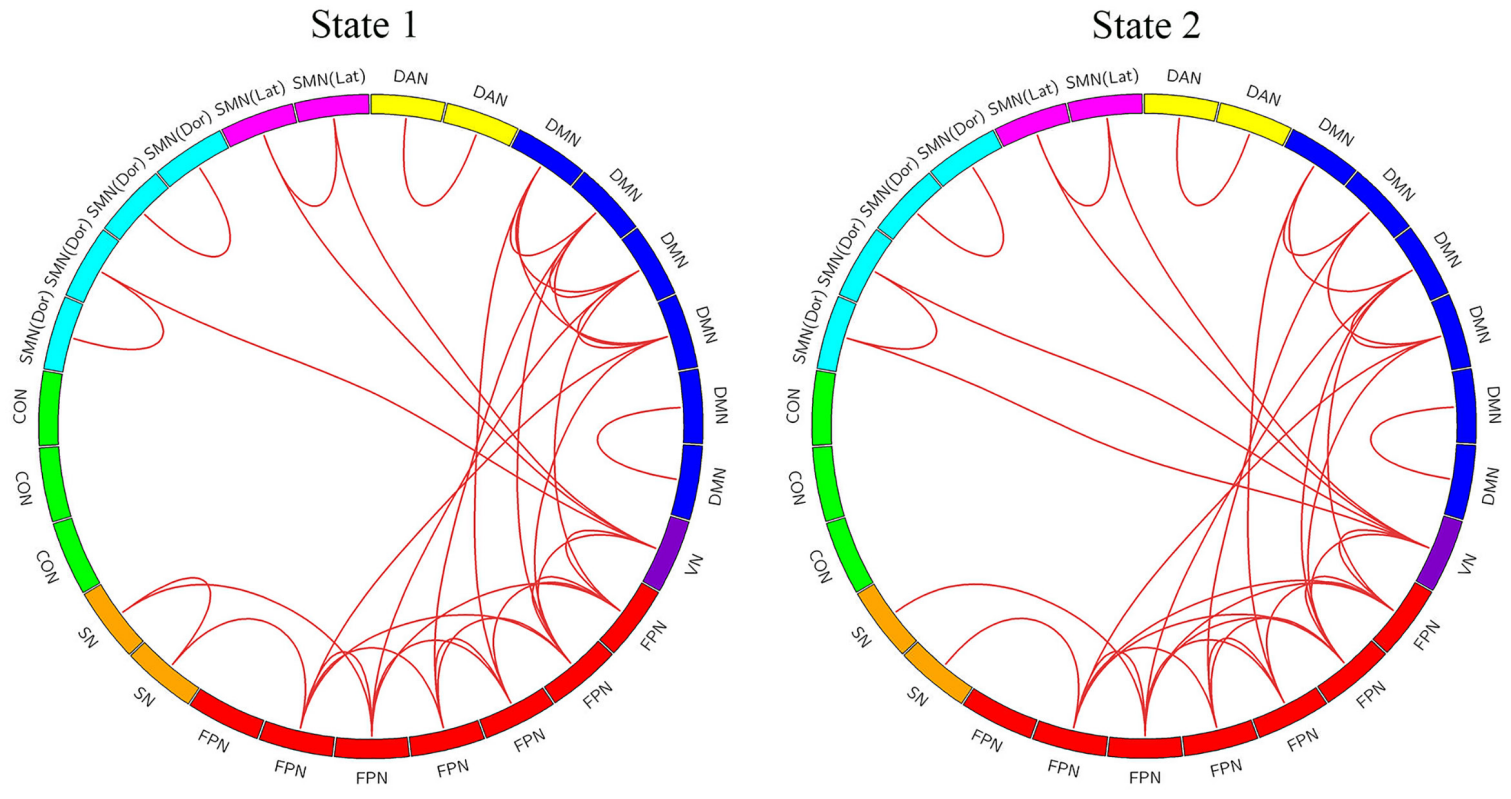
The top 10% of the strongest FC of all subjects. CON, cingulo‐opercular network; DAN, dorsal attention network; DMN, default mode network; FPN, frontoparietal network; HC, healthy controls; OSA, obstructive sleep apnea; SMN (Dor), somatomotor network‐dorsal; SMN (Lat), somatomotor network‐lateral; SN, salience network; VN, visual network.

**FIGURE 3:**
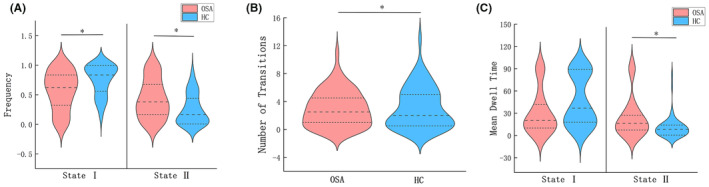
Temporal properties of dynamic functional connectivity states for the OSA patients and HC groups. (A) Frequency, (B) number of transitions, and (C) mean dwell time of OSA patients and HC, between states were plotted using violin plots. Horizontal black dotted lines indicate group medians and interquartile range. *p < 0.05. HC, healthy controls; OSA, obstructive sleep apnea.

**FIGURE 4:**
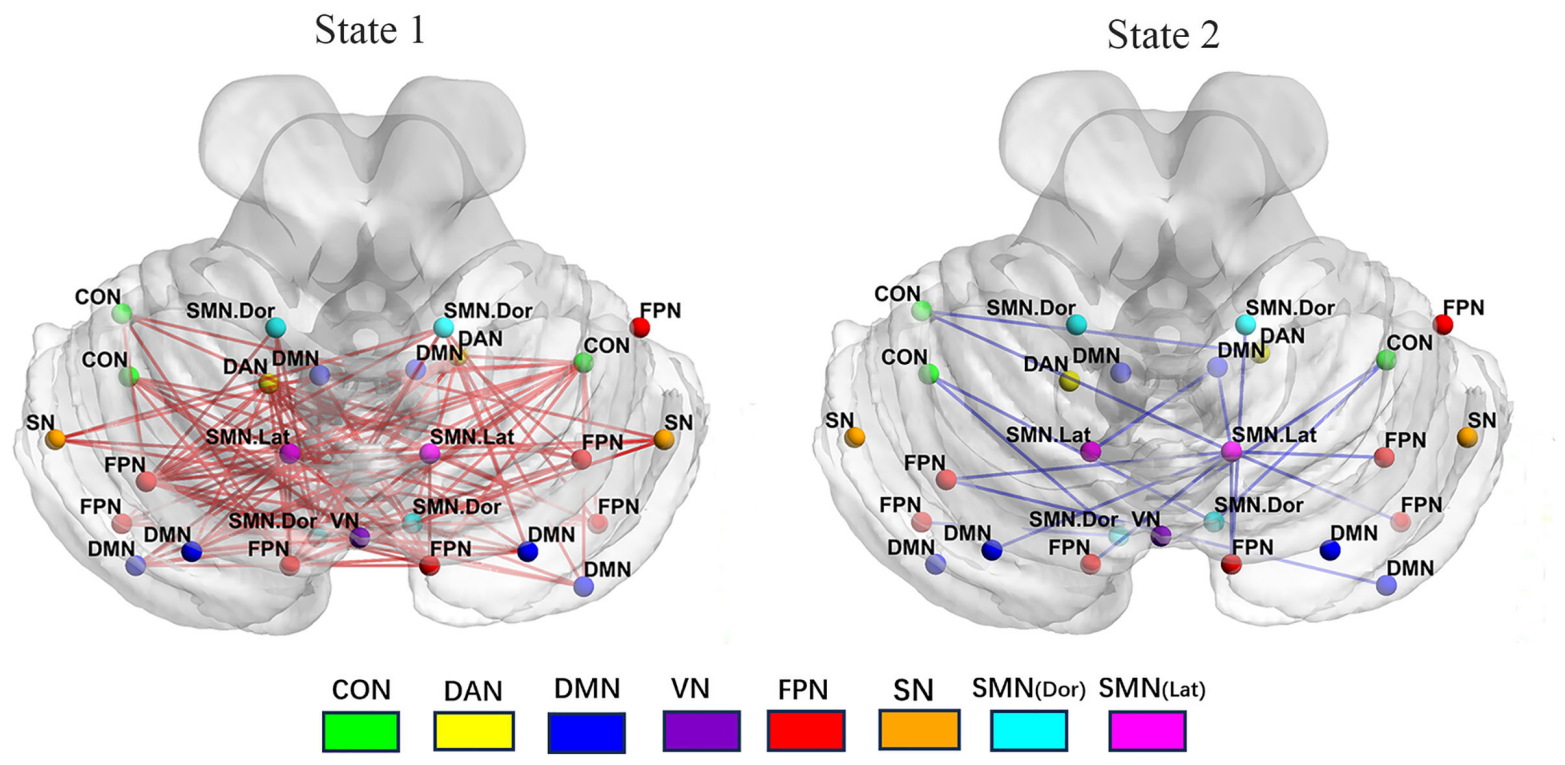
The differences in FC strength between the two groups by using NBS analysis. The red lines mean stronger connections in patients with OSA than HC in state 1, and the blue lines mean stronger connections in patients with HC than OSA in state 2. CON, cingulo‐opercular network; DAN, dorsal attention network; DMN, default mode network; FPN, frontoparietal network; HC, healthy controls; NBS, network‐based statistical; OSA, obstructive sleep apnea; SMN (Dor), somatomotor network‐dorsal; SMN (Lat), somatomotor network‐lateral; SN, salience network; VN, visual network.

The optimal number of clusters (*K* = 2) was obtained through K‐means clustering (Figure S2), and the centroid of each state was displayed in Figure 1. State 1 (66%) displayed hypoconnectivity with lower intra‐ and internetwork dFC values, manifesting more frequently in a less pronounced manner, indicative of a dissociated, low‐alertness state. Conversely, State 2 (34%) exhibited hyperconnectivity with a more tightly integrated dFC matrix. Although less frequent, it was characterized by stronger dFC values, representing an integrated state. The top 10% of the strongest FC matrix for both states in the two groups were illustrated in Figure 2 and Figure S3, aiming to characterize interactions within and between the cerebellar network. Additionally, both the top 10% of intra and internetwork exhibited robust synchronization in high‐order cognitive networks, namely, the frontoparietal network (FPN) and default mode network (DMN). The time‐varying characteristics of the two states were calculated with head movement, age, years of education, and BMI as covariates (Figure 3, Table 2). MDTD and FR were increased in OSA in state 2; the FR of OSA was significantly reduced in state 1, and NT between the two states was significantly different. The two modes of dFC in OSA and HC are shown in Figure S4. Cluster centroids of dFC for all subjects in OSA patients and HC with a sliding window length of 30 TR and steps of 1 or 2 TR (Figure S5). NBS analysis revealed differences in dFC between 2 states (*p*_component_ = 0.0001, *p*_edge_ = 0.005; Figure 4). In state 1, OSA patients exhibited enhanced internetwork FC compared to HC. While in state 2, OSA patients showed reduced FC in the somatomotor network (SMN) and FPN relative to HC.

**TABLE 2:** The temporal properties of dFC states with 50 TR window size in OSA patients and HC.

	State	OSA	HC	*p*‐value	*t*‐value
MDTD	1	31.050 ± 29.373	46.142 ± 33.233	0.995	0.007
	2	24.128 ± 26.746	10.666 ± 14.216	0.003*	2.98
FR	1	0.569 ± 0.327	0.751 ± 0.256	0.025*	−2.272
	2	0.431 ± 0.327	0.249 ± 0.256	0.025*	2.272
NT		2.983 ± 2.541	3.083 ± 2.942	0.015*	−2.478

*Note*: *p < 0.05, which was considered statistically significant, data are presented as the mean ± SD. With head movement, age, years of education, and BMI as covariates.

Abbreviations: dFC, dynamic functional connectivity; FR, frequency; HC, healthy controls; MDTD, mean dwell time of dFC; NT, number of transitions; OSA, obstructive sleep apnea.

### 3.3 Hidden Markov model validation analysis results

Two hidden states of all subjects were selected and guided by different dFC window analyses (Figure S6). The hidden state 1 aligned with state 2 of dFC, whereas the hidden state 2 matched state 1 of dFC. Characteristics of the HMM with head motion, age, years of education, and BMI as covariates are presented in Figure S7 and Table S2. The HMM outcomes predominantly implicated cognitive functional states represented by the highly interconnected DMN and FPN brain FC networks. OSA patients persisted in hidden state 1 for longer durations and were more difficult to transfer to other states. The MDTH and SR showed marked dependability, with increased MDTH in hidden state 1, observed in OSA patients, aligning with the dFC perspective. Supplementary figures visualize the HMM state characteristics and connectivity patterns for each subject (Figures S8 and S9).

### 3.4 Cerebrocerebellar results

**FIGURE 5:**
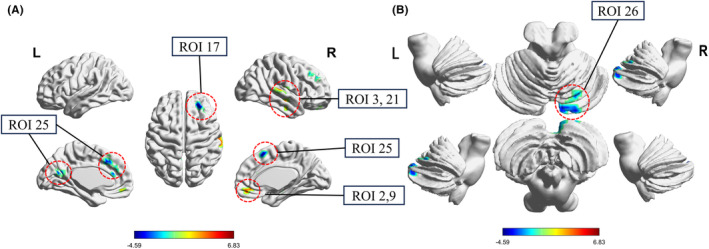
Altered dynamic functional connectivity variability between statistically significant differences cerebellar seed points of 27 regions of interest and cerebrum (A) as well as the cerebellum (B).

The variability of dFC, based on 27 cerebellar seed regions, exhibited an increase in variability between ROIs 2, 9 and the right medial orbitofrontal cortex (MOFC), as well as ROIs 13, 21 and the right superior temporal gyrus (STG) and right medial temporal gyrus (MTG). The variability of dFC was reduced with ROI 17 and the right dorsolateral prefrontal cortex (DLPFC), ROI 25 and the left retrosplenial cortex (RSC), as well as the bilateral dorsomedial prefrontal cortex (DMPFC), and ROI 26 with the right cerebellar Crus I/II (Figure 5 and Table 3).

**TABLE 3:** The significant dFC variability differences in OSA relative to HC.

Cerebellum ROI	Altered cerebrum regions	Number of voxels	Coordinates in MNI			*t*‐value
			*X*	*Y*	*Z*	
2 (DAN)	R‐MOFC (DMN)	51	15	39	−9	4.096
9 (DMN)	R‐MOFC (DMN)	41	3	48	−12	4.036
13 (FPN)	R‐STG (LN)	66	63	−27	12	4.443
17 (FPN)	R‐DLPFC (FPN)	49	24	42	30	−4.59
21 (CON)	R‐MTG (LN)	43	42	−15	−18	4.193
25 SMN(Lat)	L‐RSC (VN)	60	−9	−54	18	−3.743
	BI‐DMPFC (DMN)	75	3	18	51	−4.106
26 SMN(Lat)	R‐Crus I and II	42	18	−84	−27	−3.773

Abbreviations: BI, bilateral; CON, cingulo‐opercular network; DAN, dorsal attention network; dFC, dynamic functional connectivity; DLPFC, dorsolateral prefrontal cortex; DMN, default mode network; DMPFC, dorsomedial prefrontal cortex; FPN, frontoparietal network; HC, healthy controls; L, left; LN, language network; MNI, Montreal Neurological Institute; MOFC, medial orbitofrontal cortex; MTG, middle temporal gyrus; OSA, obstructive sleep apnea; R, right; ROI, region of interest; RSC, retrosplenial cortex; SMN, somatomotor network‐lateral; STG, superior temporal gyrus.

### 3.5 Comparison of the variability of dynamic cerebellar topological properties

The cerebellum of OSA patients comprises a high‐performance, complex network system. Both the OSA and HC groups exhibited significant small‐world organization within the functional cerebellar connectome. OSA patients showed no significant differences in nodal attributes compared to the HC group. However, in terms of global metrics, OSA demonstrated increased Eg variability and decreased Cp variability (Table 4).

**TABLE 4:** The temporal properties of dynamic topological properties with 50 TR window size in OSA patients and HC.

	OSA (*10^−4^)	HC (*10^−4^)	*p*‐value	*t*‐value
Eg	1.066 ± 0.707	0.786 ± 0.452	0.018**	2.393
Cp	1.256 ± 0.604	1.541 ± 0.869	0.028**	−2.223

*Note*: **p < 0.05, which was considered statistically significant, data are presented as mean ± SD. With head movement, age, years of education, and BMI as covariates.

Abbreviations: Cp, characteristic path length; Eg, global efficiency; HC, healthy controls; OSA, obstructive sleep apnea.

### 3.6 Correlation analysis

**FIGURE 6:**
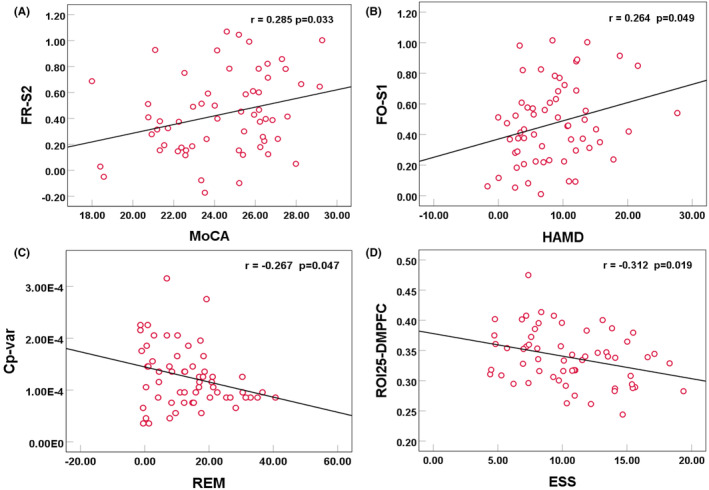
Correlation analysis of (A) dFC, (B) HMM, (C) dGT metrics, and (D) cerebrocerebellar dFC alterations with clinical scale indicators in OSA patients. For full details, refer to Table S3. Cp‐var, the variance of characteristic path length; dFC, dynamic functional connectivity; dGT, dynamic graph theory; DMPFC, dorsomedial prefrontal cortex; ESS, Epworth Sleepiness Scale; FO, fractional occupancy; FR, frequency; HAMD, Hamilton Depression Scale; HMM, Hidden Markov Model; MoCA, Montreal Cognitive Assessment; OSA, obstructive sleep apnea; REM, rapid eye movement; ROI, region of interest; S1/2, state1/2; SR, switching rate.

The MDTD positively correlated with REM and negatively correlated with HAMD and MoCA in state 1. In contrast, MDTD positively correlated with HAMD in state 2. The correlation between the FR of dFC and MoCA switched from negative in state 1 to positive in state 2. The FO showed a positive correlation with MoCA and HAMD in state 1 but was reversed in state 2. The MDTH across both states and NT of dFC, SR of HMM, and Cp variance of dGT all showed a negative correlation with REM. Furthermore, dFC variability between ROI 21 and MTG, ROI 25 and RSC, as well as ROI 25 and DMPFC, negatively correlated with SE and ESS. These correlations were shown in Figure 6 and detailed in Table S3.

## 4 DISCUSSION

In this study, cerebellar seed points were utilized to characterize the cerebellar‐whole brain network based on dFC as well as topological changes between OSA patients and HC. The HMM temporal parameters demonstrated the reliability of the dFC parameters and dynamic temporal traits. Key findings include two repetitive dFC patterns in the instinctive brain. State 1 represents a “baseline state” characterized by inefficient functional integration, while state 2 reflects a state of preparedness to perform high‐alert tasks. And FR and MDTD were prolonged in state 2 under OSA. Additionally, the NBS analysis revealed that the connectivity strength was primarily concentrated on the DMN and frontoparietal network (FPN).

### 4.1 Dynamic functional connectivity of cerebellar networks

According to previous studies, OSA patients spend more time in the weak connection state.^39^ The brain network remodeling in OSA patients may indicate a trend toward attenuated interactions among brain regions, challenging the maintenance of a baseline state for prolonged durations. To perform various high‐order cognitive functions, key nodes were postulated to strengthen connections, consequently increasing active state frequency and enabling a transition to state 2. On the other hand, chronic hypoxia prevents cerebellar maintenance of enhanced vigilance despite initial success, potentially due to enduring exposure. Substantially increased state 2 connectivity variability may enable sustained informational capacity and early‐phase selective neurocognitive resilience in OSA via dynamic connectivity. However, prolonged dependence on this pathway impeded effective state switching and led to network stagnation. Moreover, OSA was surmised to be highly oscillatory in nature, with its dynamic network traits potentially subserving an adaptive role.^40^, ^41^, ^42^ State transition and temporal metrics exhibited negative correlations with REM, implicating activation of brain plasticity mechanisms by OSA patients to stabilize states and preserve the REM state to bolster global brain network function. Furthermore, select dynamic metrics portrayed negative correlations with HAMD and MoCA scores. Prior studies have demonstrated that compromised sleep quality impacts emotional stability, engendering depressive states,^43^ which may constitute prodromal manifestations of forthcoming cognitive decline.^44^

Imbalances in functional segregation and integration may underpin cognitive, emotional, behavioral, and sleep dysfunctions in OSA. In this study, heightened within‐network modularity and cross‐network synergistic enhancement may constitute vital mechanisms driving early impairments. Persistent DMN activation in regulating other networks may explain the difficulty of disengaging from state 2 and maintaining stability.^45^, ^46^ Within high‐order cognitive networks, the DMN can provide scaffolding support to the FPN, which regulates cognitive control and goal‐directed behaviors, thereby facilitating working memory development.^47^ The SN serves as a cognitive activity switching hub, coordinating transitions between the DMN and DAN.^48^ In low‐level cognitive networks, the SMN and DMN are complementary in regulating emotion, cognition, working memory, and locomotion. Decoupling between the SMN and DMN predicts improved cognitive performance.^49^ OSA patients exhibit impairments in reaction time, sustained attention, visuospatial processing, and executive functions,^50^ and alterations in visual information processing may engender cognitive decline in OSA.^51^ The VN provides sensory guidance for the SMN, and their synergy is crucial for motor coordination in OSA patients. The increased connectivity may be used to compensate for the imbalance in motor coordination in OSA patients.^52^ Therefore, imbalances between networks in which the cerebellum plays a key coordinating role between upper and lower networks, possibly through the DMN, modulate cognitive, emotional, and sleep functions in OSA, thereby maintaining overall systemic stability.

### 4.2 Dynamic cerebrocerebellar functional connectivity variability

Among regions exhibiting increased dFC variability with the cerebellum, the MOFC constitutes part of the DMN. Alterations in the DMN may underlie cognitive deficits in OSA patients.^33^, ^53^ The DMN mitigates cognitive deficiencies by modulating connections and activating peripheral cognitive networks.^4^ Moreover, the impacts of OSA severity and nocturnal oxygen desaturation on language fluency,^54^ increased variability in the language network (LN), and cerebellar connectivity imply enhanced coordination therein to preserve language abilities in OSA patients. Stability in the left FPN, also participating in language, suggests possible LN reorganization and less reliance on left frontoparietal regions. In contrast, reduced right FPN (responsible for directional attention and spatial localization) variance indicates atypical neural signaling in right frontoparietal areas affected in OSA.^55^ Furthermore, in brain regions exhibiting decreased FC, the VN and SMN are vital for motor control and learning. Reduced connectivity between them could underpin cognitive deficits by disrupting visual processing.^37^

The DMPFC constitutes a key node within the DMN involved in sleep regulation.^56^ The cognitive cerebellum, which supports executive functions and attentional shifts, receives input from the sensory cortex and coordinates SMN activity.^57^, ^58^ Studies have indicated OSA patients have elevated motor vehicle accident risk,^59^, ^60^ likely linked to SMN dysfunction, impaired spatial cognition due to FPN and DMPFC disturbances leading to attentional lapses and concentration issues, as well as SMN‐VN interaction abnormalities and decreased driving coordination ability.

Interestingly, reduced functional connectivity variability between the cerebrocerebellar VN and SMN was attenuated, which contrasts with the changes in the cerebellar network. Disruption in cerebrocerebellar FC has been reported in various neurological and neurocognitive disorders.^61^ To respond to the potential damage of OSA and self‐regulation of cerebrocerebellar network disconnection, the brain prioritizes utilizing resources to maintain and improve the capacity of the cerebellar network for independent visuomotor information processing. Specifically, the cerebellum may act to internally compensate for OSA‐related pathophysiology, while extra cerebellar regions display asynchronous, decoupled activity patterns. In general, OSA patients may exhibit prioritization limitations in brain functional networks, allocating more resources to high‐order cognition, potentially as compensation. This coordination of high‐order networks such as the DMN may disrupt interactions among basic cognitive task networks responsible for perception, attention, and memory. The resulting imbalance could impair low‐level networks while sustaining high‐order functions.

### 4.3 Dynamic graph theory

The current findings substantiate that OSA patients display small‐world properties and modified topological indices in their cerebellar connectomes.^4^, ^15^ The adjusted variations in Eg and Cp imply that the brain's functional network operates at a decreased level of transmission efficiency and regulation. The processing efficiency of the cerebellar network is diminished due to attenuated variability in the transmission capacity of neighboring nodes and the delayed responsiveness of adjacent nodes in the cerebellum of OSA patients. This reduction in fault tolerance predisposes the network to disruption and injury, thus potentially constituting a mechanism underlying cognitive deficits. At this time point, between‐group fluctuations within the functional brain network declined over time, suggesting OSA patients were more prone to persist in a stable network state. Consequently, connections between distant brain regions increased, with more frequent network regulation occurring, enhancing Eg variability to improve information processing efficiency. Such optimization may facilitate cerebellar integration and cognitive flexibility, permitting additional interactions between high‐order cognition and sensory processing networks to enhance information transfer efficiency. However, these alterations represented transient rather than optimally stable conditions, with inefficient transitional states persisting.

Longitudinally, such functional decoupling may precipitate cognitive decline. This could be attributed to nodes being confined to localized regions, enabling easier stabilization via regional modulation, whereas global attributes represent overall network characteristics with complex regulatory schemes beyond brain plasticity. Nevertheless, OSA‐provoked compensatory mechanisms can maintain relative nodal stability. Moreover, an inverse correlation was discovered between Cp and REM, potentially indicative of stability preservation through self‐optimization combined with REM augmentation to mitigate functional impairments from unstable connections. Intrinsic stabilization efforts, in addition to recognizing constrained self‐organization capacities, highlight the necessity of REM sleep for sustaining function amidst OSA disruption.

## 5 LIMITATION

This study has several limitations. First, dFC network calculations only examined gray matter; future efforts should optimize methodologies to incorporate white matter data. Additionally, we exclusively investigated FC networks, and examining structural connectivity network topological changes is recommended moving forward. A further limitation of our study is the potential variability in disease duration among individuals with OSA. Future studies should investigate the impact of the impact of disease duration on cerebellar and FC changes in OSA patients. Furthermore, all the subjects enrolled in this study were adult males. Future studies should expand the sample size and include more female and pediatric patients to improve the generalizability of the results. Finally, despite employing a voxel‐wise threshold of *p* < 0.005 and cluster‐level *p* < 0.05, it is essential to acknowledge inherent limitations. The utilization of a lenient statistical threshold may heighten the likelihood of false positives, necessitating judicious interpretation.

## 6 CONCLUSION

In summary, through dFC and dGT analyses, we propose that interactions between multiple high‐order and low‐level cognitive circuits modulated by DMN are involved in regulating language, cognition, sleep, emotion, and motor function in OSA. Furthermore, OSA patients may maintain cognitive stability through functional coordination and compensation between distinct brain areas and networks. Our results may have novel perspectives on endogenous brain activity in OSA and elucidate the neural mechanisms underlying cognitive deficits.

## AUTHOR CONTRIBUTIONS

DP guided and designed the MRI experiment. LL and TL analyzed and discussed the ideas of the manuscript. LL wrote the manuscript. YL organized the results. MA and YS processed the data. YS and XL analyzed the resting‐state fMRI data. LZ, LH, YL, and YD collected the resting fMRI data and applied it for ethics approval. HL and DP reviewed and revised the manuscript. All authors contributed to the article and approved the submitted version.

## FUNDING INFORMATION

This study was supported by the National Natural Science Foundation of China (Grant Nos. 81860307, 81960609), Natural Science Foundation Project of Jiangxi Province, China (Grant Nos. 20202BABL216036, 20181ACB20023), Education Department Project of Jiangxi Province, China (Grant No. GJJ190133), Department of Health Project of Jiangxi Province, China (Grant No. 202210211). Science and technology plan project of Jiangxi Administration of Traditional Chinese Medicine (Grant No. 2023A0278), Clinical Research Center For Medical Imaging In Jiangxi Province (No. 20223BCG74001), and Jiangxi Provincial Department of Graduate Innovation Foundation (Grant No. YC2023‐B077).

## 7 CONFLICT OF INTEREST STATEMENT

The authors have no financial conflicts of interest to declare.

## Supporting information


Appendix S1.

Figure S1.

Figure S2.

Figure S3.

Figure S4.

Figure S5.

Figure S6.

Figure S7.

Figure S8.

Figure S9.

## Data Availability

The original data of this paper are provided by the author, there is no unnecessary retention, and the data set is still being supplemented, which is related to the follow‐up longitudinal study of our research group.
