# Supplementary material for: Abnormal dynamic functional connectivity and topological properties of cerebellar network in male obstructive sleep apnea
Source: CNS Neurosci Ther. 2024 Jun 3;30(6):e14786. doi: 10.1111/cns.14786 (PMC11145370; doi:10.1111/cns.14786)
Supplement: Supplementary file 1 — Appendix S1. [file CNS-30-e14786-s002.docx]

**Supplementary Materials**

**Methods**

**Supplementary method 1**

Each participant completed a standardized series of neuropsychological assessments before scanning. The Epworth Sleepiness Scale (ESS) evaluates daytime sleepiness (scores ranging from 0-24; > 6 indicates drowsiness, >11 excessive indicates drowsiness, and＞16 indicates dangerous drowsiness). The Pittsburgh Sleep Quality Index (PSQI) was used to assess subjective sleep quality and disturbances over the past month (scores range from 0-21; higher scores indicate poorer quality). The Montreal Cognitive Assessment (MoCA) was administered to assess cognitive function across eight domains. The MoCA generates a total score ranging from 0-30, with <26 indicating mild cognitive impairment (MCI) ^1^. The Hamilton Anxiety Scale (HAMA) and Hamilton Depression Scale (HAMD) are used to evaluate anxiety and depressive symptoms. HAMA scores range from 0-56, where >7 denotes anxiety and >29 severe anxiety. HAMD scores range from 0-52, where >7 indicates depression, >17 mild to moderate depression, and >24 indicates severe depression.

**Supplementary method 2**

The fMRI data were subjected to standard preprocessing procedures as follows: (1) The first 10 time points of each fMRI scan were discarded to minimize the impact of magnetic saturation and scanner noise. (2) Slice timing correction and three-dimensional head motion correction were subsequently applied on the remaining 230 volumes. The data would be eliminated if a participant's maximal head movement was more than 1.5 mm in any direction or if the angle on any axis was rotated by more than 1.5° across the time series. (3) Co-register the T1-weighted structural image of each subject with the average value of the realigned EPI image. (4) Use the new segmentation method in SPM12 to segment the transformed structural image into white matter, gray matter, and cerebrospinal fluid. (5) Normalize the functional data space to the Montreal Neurological Institute (MNI) template with 3×3×3mm^3^ voxels. (6) Regresse out nuisance covariates including 24 head motion parameters (including 6 head motion parameters, 6 head motion parameters one time point before, and the 12 corresponding squared items), global mean signals, white matter, and cerebrospinal ﬂuid signals. (7) Time bandpass filtering (0.01–0.08 Hz) was used to avoid the effects of low-frequency drift, physiological high-frequency noise, and cardiac fluctuations.

**Supplementary method 3**

**Descriptions of the graph measures**

| **Graph measure** | **Definition** | **Meaning** |
| --- | --- | --- |
| **Clustering coefficient** | quantifies the ratio of existing connections between the node's neighbors over all their possible connections | a measure of the prevalence of clustered local connectivity |
| **Characteristic path length** | the average shortest path length between all pairs of nodes in a network | a measure of the overall routing efficiency of the network |
| **Gamma** | characterizes the extent of network segregation by measuring the ratio between observed edges and maximum possible edges | higher gamma indicates a stronger clustering tendency |
| **Lambda** | delineates integration efficiency by computing the mean distance between all vertex pairs | shorter lambda corresponds to a higher global reach |
| **Sigma** | gauges the balance of segregation and integration by normalizing the ratio of gamma and lambda against randomized networks | larger sigma signifies the concurrent existence of dense local clustering and global reach |
| **Global efficiency** | measures the average inverse shortest path length between all node pairs in the network | characterizes the capacity for parallel information transfer across the whole network |
| **Local efficiency** | quantifies the efficiency of information exchange within the neighborhood of each node upon removal of that node | depicts the prevalence of clusters and fault tolerance arising from the redundancy of local connections |
| **Nodal degree** | the number of edges directly connected to a node | encapsulates its extent of direct connections and local influence within the network |
| **Nodal efficiency** | characterizes the mean geodesic distance from a node to all other nodes | quantifies the capability to propagate information in parallel while reaching other nodes efficiently |
| **Nodal betweenness** | signifies the fraction of shortest paths between node pairs that traverse through the node | measures the potential control over information flow as an intermediary broker between distant nodes |

**Results**

| **Table S1: Coordinates and functional networks of 27 cerebellar seed regions^2^** | | | | |
| --- | --- | --- | --- | --- |
| **Networks** | **ROI** | **Peak MNI coordinate** | | |
|  |  | **X** | **Y** | **Z** |
| **Cingulo-Opercular Network** | 1 | 32 | -49 | -51 |
|  | 20 | -34 | -42 | -44 |
|  | 21 | -33 | -51 | -50 |
| **Dorsal Attention Network** | 2 | -13 | -52 | -50 |
|  | 3 | 14 | -48 | -52 |
| **Default Mode Network** | 4 | -32 | -78 | -38 |
|  | 5 | 32 | -81 | -38 |
|  | 6 | -24 | -76 | -28 |
|  | 7 | 24 | -76 | -28.01 |
|  | 8 | -5.72 | -50.8 | -40.84 |
|  | 9 | 8 | -50 | -40 |
| **Visual Network** | 10 | 0 | -74 | -25 |
| **Frontoparietal Network** | 11 | -10 | -78 | -28 |
|  | 12 | 10 | -78 | -28 |
|  | 13 | -34 | -72.01 | -48 |
|  | 14 | 34 | -72 | -48 |
|  | 15 | -30.5 | -66 | -30 |
|  | 16 | 31.68 | -62.83 | -30.4 |
|  | 17 | 40 | -44 | -38 |
| **Salience Network** | 18 | 43.5 | -60 | -30 |
|  | 19 | -43.5 | -60 | -30 |
| **Somatomotor Network-Dorsal** | 22 | -6 | -74 | -42 |
|  | 23 | 7.5 | -72 | -39 |
|  | 26 | -12 | -44 | -18 |
|  | 27 | 12 | -44 | -18 |
| **Somatomotor Network-Lateral** | 24 | -10 | -62 | -18 |
|  | 25 | 10 | -62 | -18 |

***Abbreviations:*** ROI**,** region of interest; MNI, Montreal Neurological Institute.

| **Table S2 The properties of** **HMM states in OSA patients and HC** | | | | | |
| --- | --- | --- | --- | --- | --- |
|  | **State** | **OSA** | **HC** | ***p-*value** | **t-value** |
| **MDTH** | 1 | 20.517±37.153 | 10.447±9.398 | **0.021*** | 2.335 |
|  | 2 | 15.467±14.459 | 20.037±15.935 | 0.786 | -0.273 |
| **FO** | 1 | 0.471±0.274 | 0.385±0.248 | 0.384 | 0.875 |
|  | 2 | 0.529±0.274 | 0.615±0248 | 0.384 | -0.875 |
| **SR** |  | 0.070±0.028 | 0.069±0.023 | **0.009 *** | -1.709 |

# *Abbreviations:* HMM, hidden Markov model; OSA, obstructive sleep apnea; HC, healthy controls; MDT, mean dwell time of HMM; FO, fractional occupancy; SR, switching rate.

| **Table S3: Significant correlation between dFC, HMM, dGT, cerebrocerebellar dFC alterations and** **clinical scale indicators in the OSA group** | | | | |
| --- | --- | --- | --- | --- |
|  | **Factor1** | **Factor2** | **r value** | ***p*-value** |
| **dFC** | MDTD-S1 | REM | 0.272 | 0.043 |
|  | MDTD-S1 | MoCA | -0.309 | 0.021 |
|  | MDTD-S1 | HAMD | -0.310 | 0.020 |
|  | MDTD-S2 | HAMD | 0.298 | 0.026 |
|  | FR-S1 | MoCA | -0.285 | 0.033 |
|  | FR-S2 | MoCA | 0.285 | 0.033 |
|  | NT | REM | -0.307 | 0.021 |
| **HMM** | FO-S1 | MoCA | 0.345 | 0.009 |
|  | FO-S1 | HAMD | 0.264 | 0.049 |
|  | FO-S2 | MoCA | -0.345 | 0.009 |
|  | FO-S2 | HAMD | -0.264 | 0.049 |
|  | MDTH-S1 | N2 | -0.342 | 0.010 |
|  | MDTH-S2 | N1 | -0.292 | 0.029 |
|  | MDTH-S2 | MoCA | -0.264 | 0.050 |
|  | SR | REM | -0.273 | 0.042 |
| **dGT** | Cp-var | REM | -0.267 | 0.047 |
| **cerebrocerebellar dFC alterations** | ROI21- MTG | SE | -0.319 | 0.016 |
|  | ROI25- RSC | SE | -0.276 | 0.040 |
|  | ROI25-DMPFC | ESS | -0.312 | 0.019 |

***Abbreviations***: dFC, dynamic functional connectivity; HMM, Hidden Markov Model; dGT, dynamic graph theory; OSA, obstructive sleep apnea; MDTD, mean dwell time of dFC; MDTH, mean dwell time of HMM; S1/2, state1/2; FR, frequency; NT, number of transitions; FO, fractional occupancy; SR, switching rate; Cp-var, the variance of characteristic path length; ROI, region of interest; MTG, middle temporal gyrus; RSC, Retrosplenial cortex; DMPFC, dorsomedial prefrontal cortex; REM, rapid eye movement; SE, sleep efficiency; MoCA, Montreal Cognitive Assessment; HAMD, Hamilton Depression Scale; ESS, Epworth Sleepiness Scale.

**Figures and Figure Legends**

**
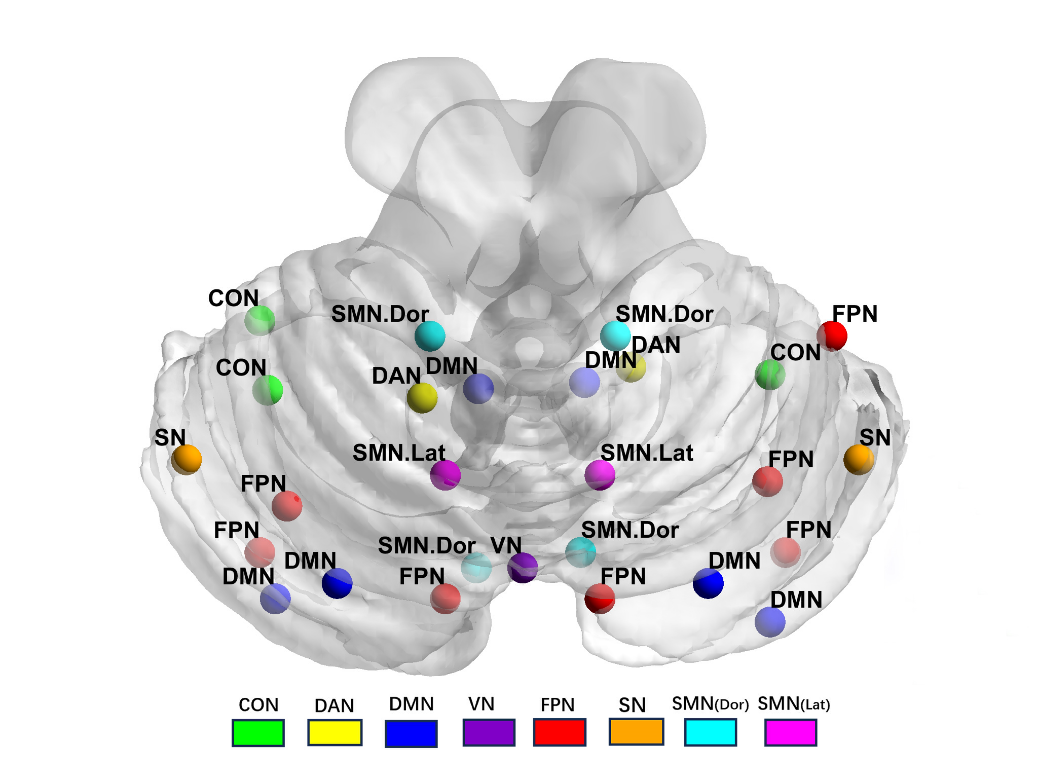
**

**Figure S1：Twenty-seven cerebellar seed points based on the Seitzman atlas^2^, including 8 networks. *Abbreviations*:** DAN, dorsal attention network; DMN, default mode network; VN, visual network; FPN, frontoparietal network; SN, salience network; CON, cingulo-opercular network; SMN(Dor), somatomotor network-dorsal; SMN(Lat), somatomotor network-lateral.

**
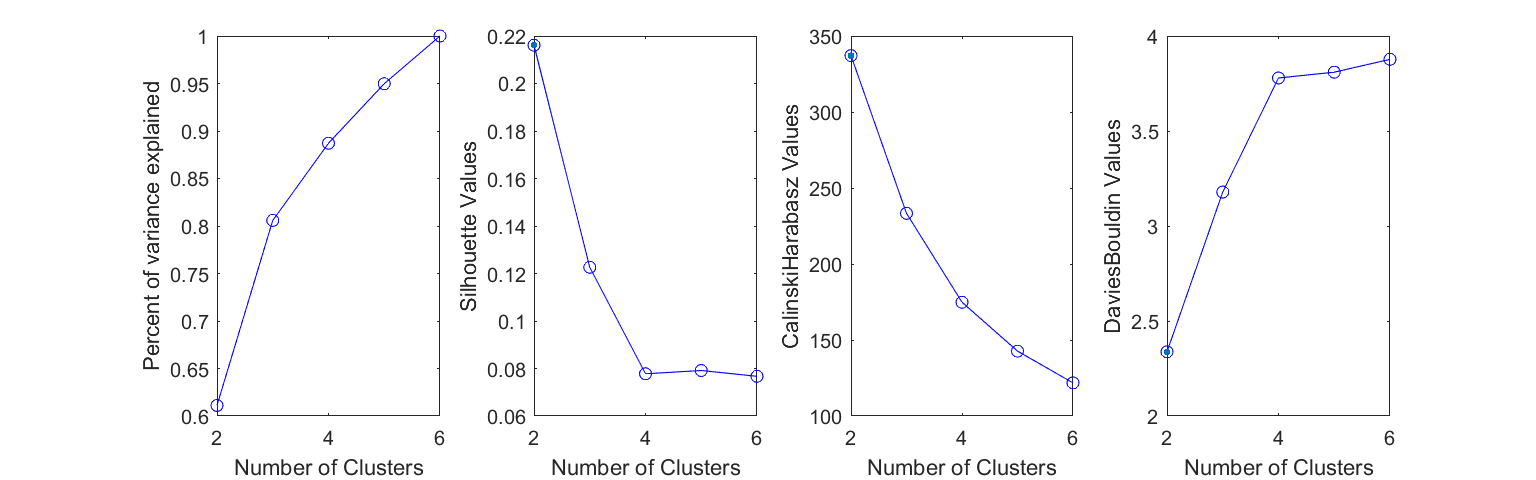
**

# Figure S2: The optimal K number according to K-means clustering analysis.

# Note: According to the percent of variance explained, Silhouette, CalinskiHarabasz, and DaviesBouldin statistics, two clusters were found in this study.

#
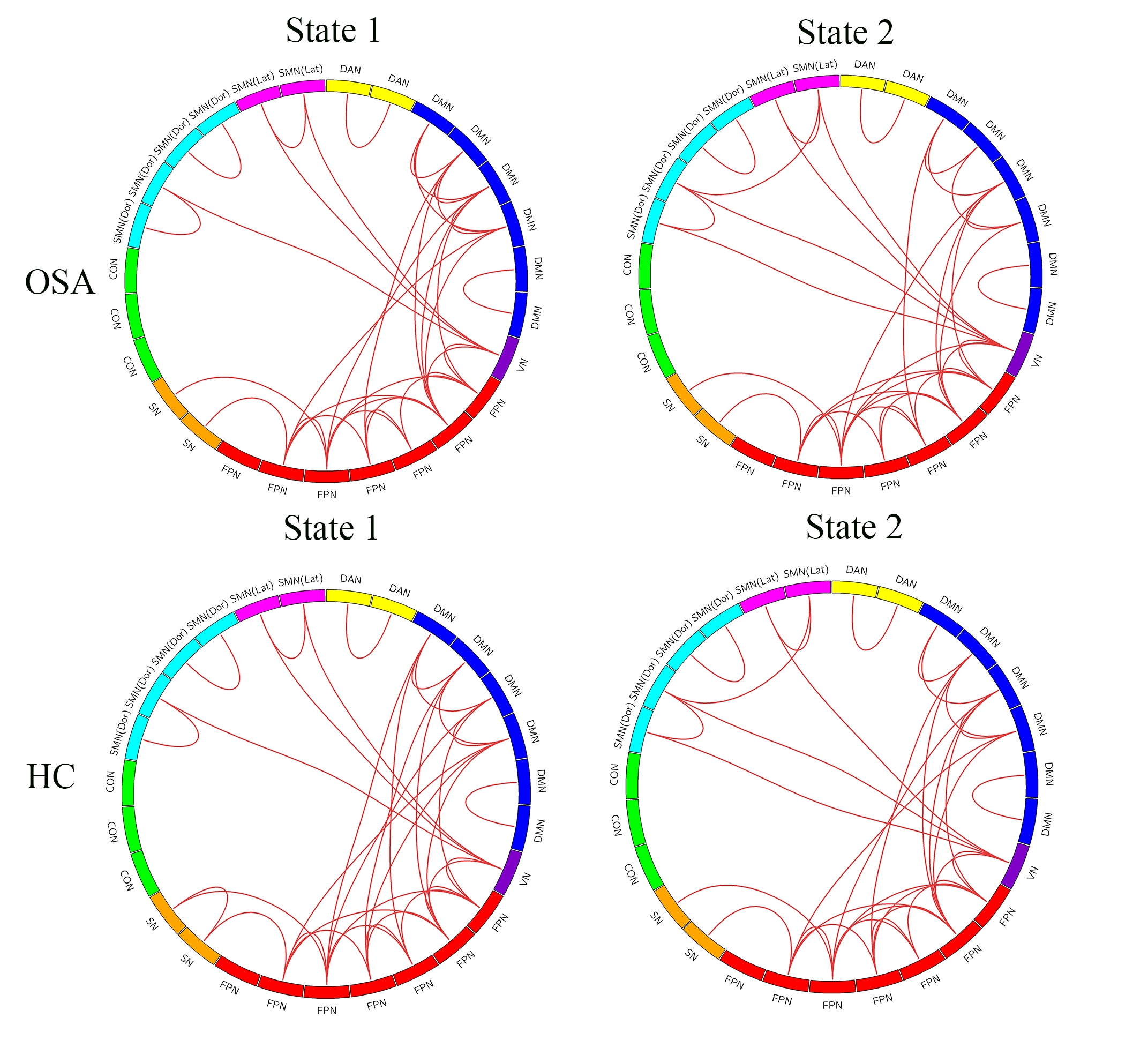


# Figure S3: The top 10% of the strongest FC in each state of OSA patients and HC. *Abbreviations:* OSA, obstructive sleep apnea; HC, healthy controls; DAN, dorsal attention network; DMN, default mode network; VN, visual network; FPN, frontoparietal network; SN, salience network; CON, cingulo-opercular network; SMN(Dor), somatomotor network-dorsal; SMN(Lat), somatomotor network-lateral.

#
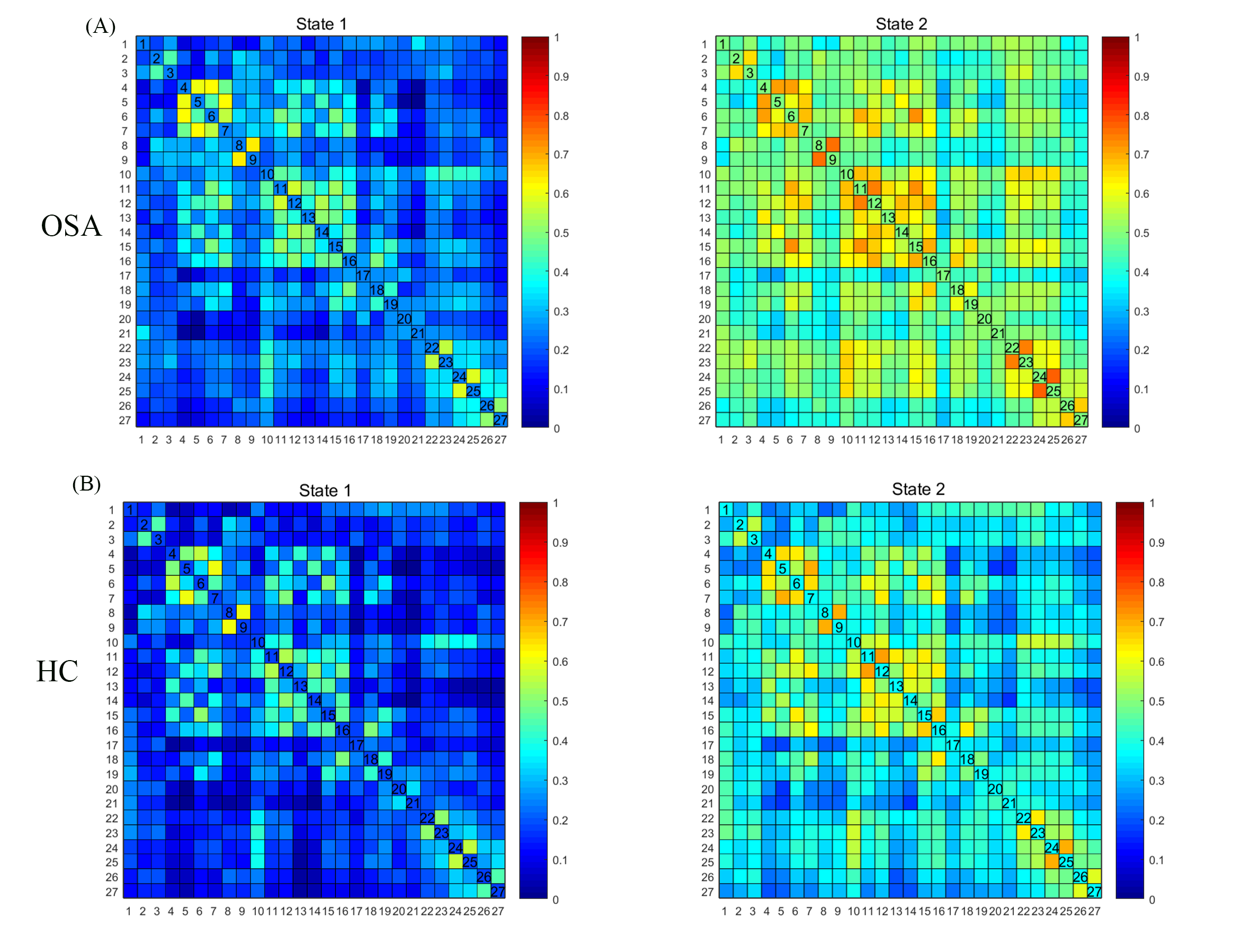
 Figure S4: Cluster centroids for each state in OSA patients and HC with sliding window length of 50 TR and steps of 2 TR. *Abbreviations*: OSA, obstructive sleep apnea; HC, healthy controls; TR, repetition time. The values ranging from 1 to 27 in the X and Y coordinates of the matrix respectively correspond to the eight distinct networks enumerated in Table 1.

#

#
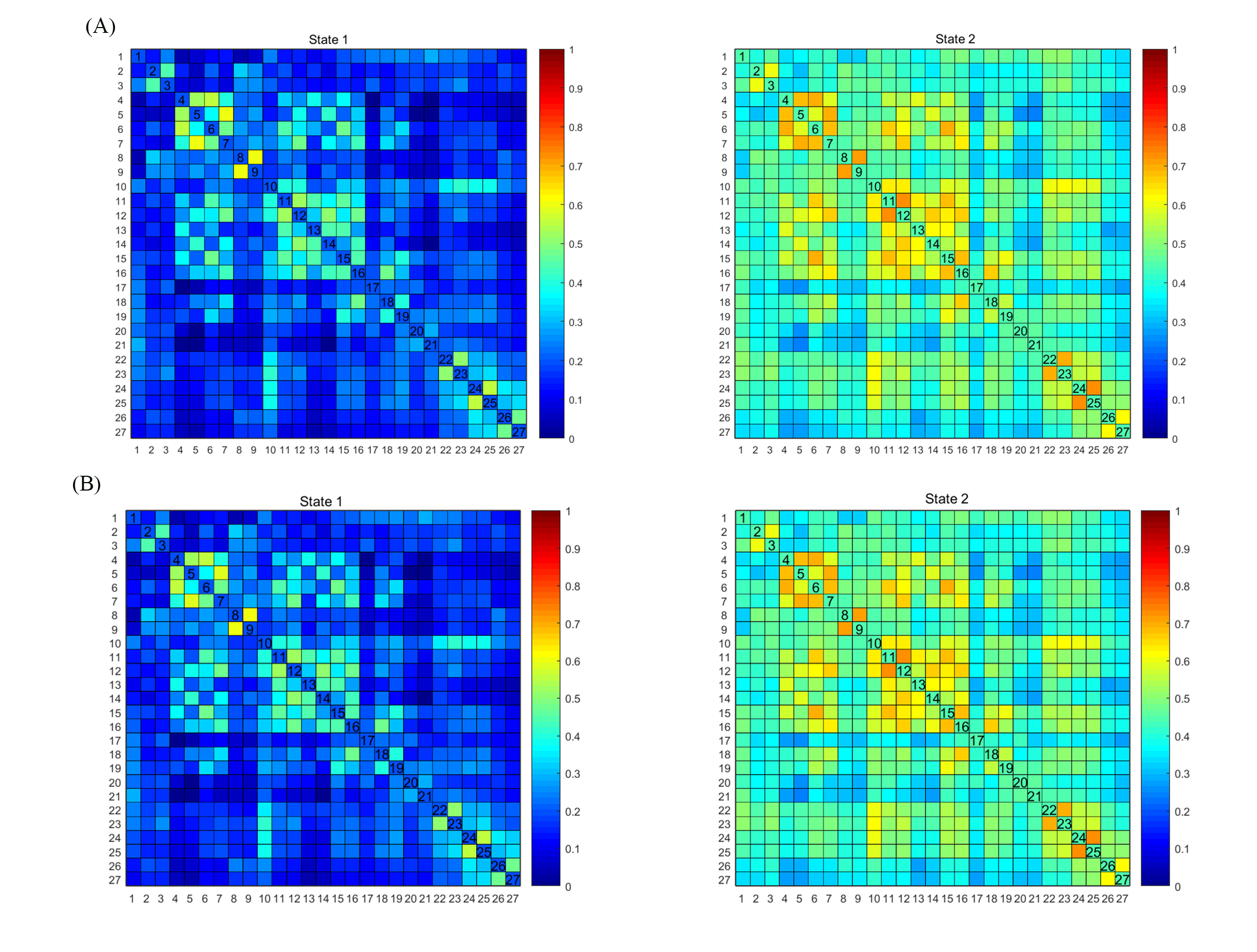


# Figure S5: Cluster centroids for each state in OSA patients and HC. (A) Sliding window length of 30 TR and steps of 1 TR; (B) Sliding window length of 30 TR and steps of 2 TR. *Abbreviations*: OSA, obstructive sleep apnea; HC, healthy controls; TR, repetition time.


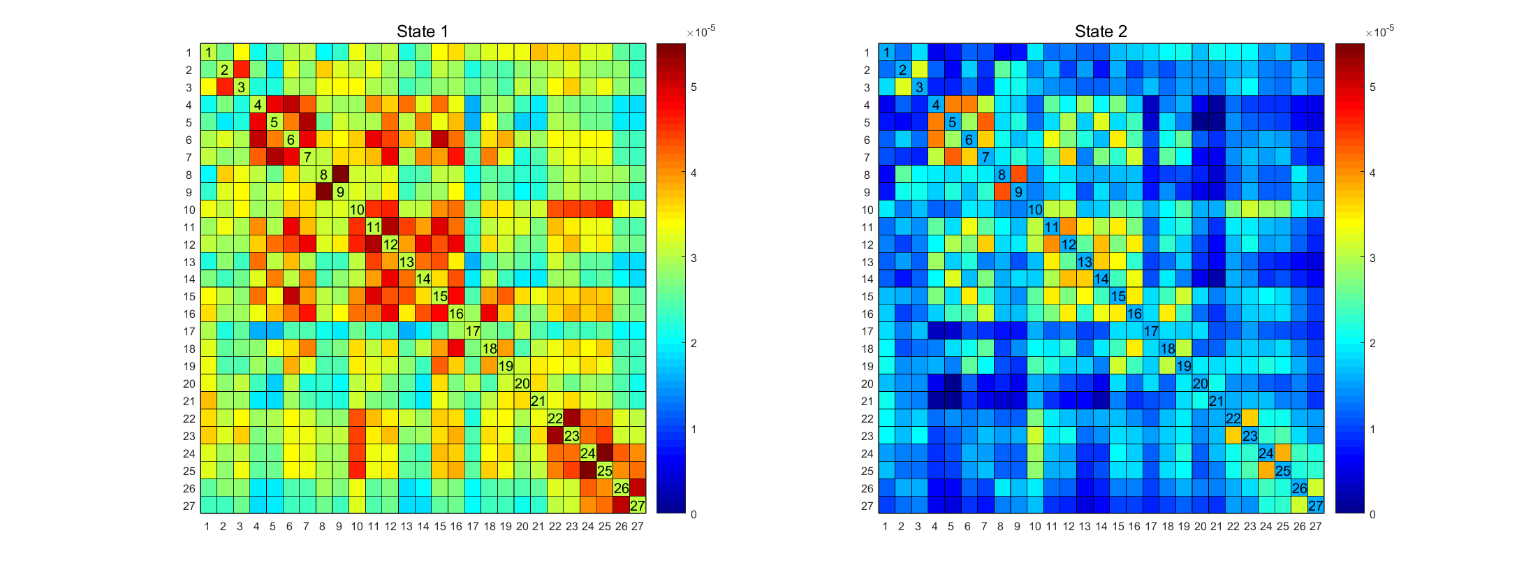
**Figure S6: Two hidden Markov model states of all subjects obtained from resting-state functional MRI data. *Abbreviations*:** OSA, obstructive sleep apnea; HC, healthy controls. The values ranging from 1 to 27 in the X and Y coordinates of the matrix respectively correspond to the eight distinct networks enumerated in **Table 1**.

#

Figure S7: Temporal properties of hidden Markov model (HMM) states for the OSA patients and HC. (A) Fractional occupancy, (B) Switching rate, and (C) Mean dwell time between states were plotted using violin plots. Horizontal black dotted lines indicate group medians and inter-quartile range. ∗*p* < 0.05. *Abbreviations*: OSA, obstructive sleep apnea; HC, healthy controls.

#

Figure S8: Diagram of hidden Markov model(HMM)

# (A) The hidden states (K=2) over 230 time points for all subjects (n=120) of the hidden Markov model. (B). The posterior probabilities (gamma values) of the two states for the first 1000 time points in the observation sequence of the hidden Markov Model.

#
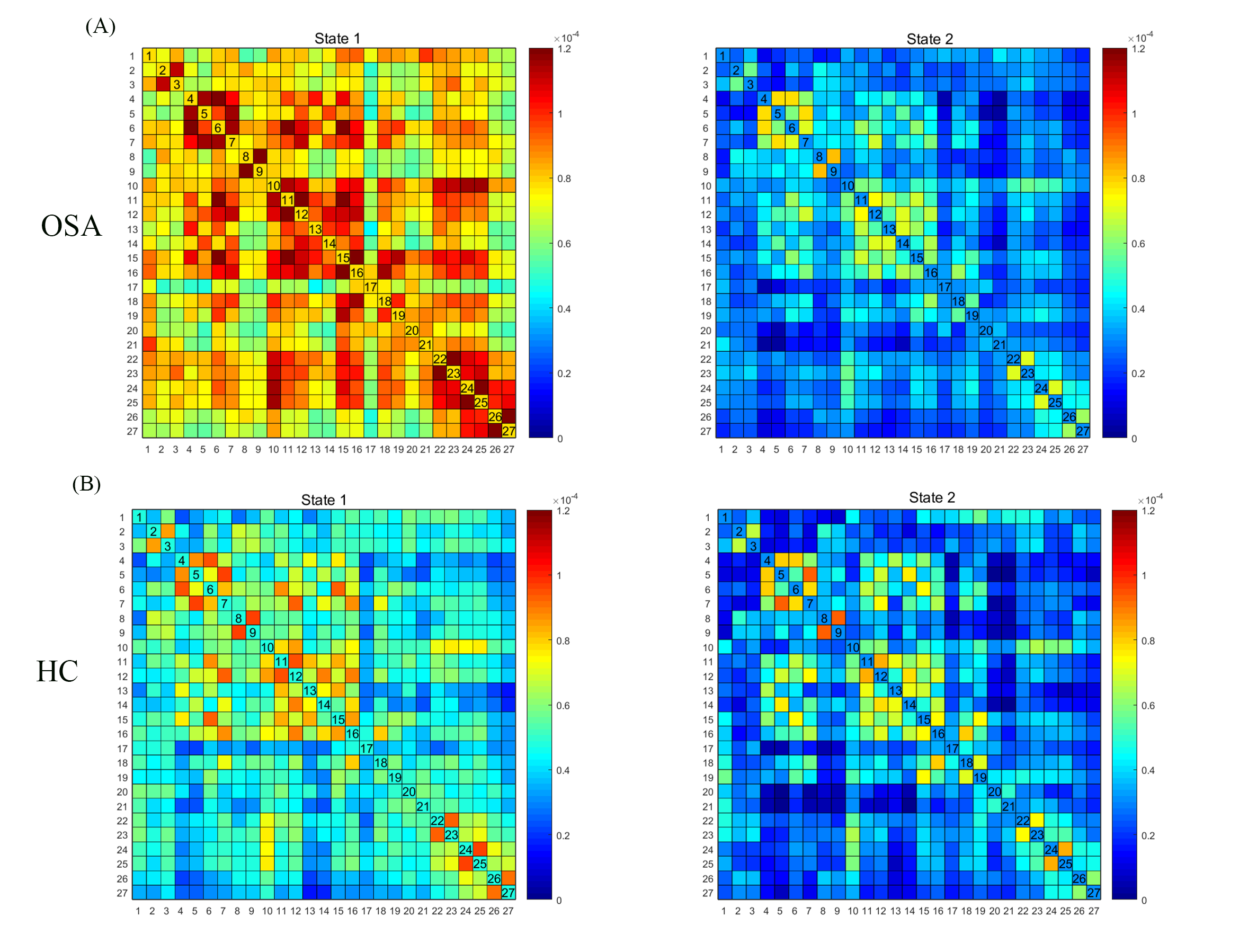
Figure S9：Two hidden Markov model states of OSA patients and HC obtained from resting-state functional MRI data. *Abbreviations*: OSA, obstructive sleep apnea; HC, healthy controls. The values ranging from 1 to 27 in the X and Y coordinates of the matrix respectively correspond to the eight distinct networks enumerated in Table 1.

**References**

1. Johansson ME, van Lier NM, Kessels RPC, Bloem BR, Helmich RC. Two-year clinical progression in focal and diffuse subtypes of Parkinson's disease. *NPJ Parkinsons Dis.* 2023;9(1):29.

2. Seitzman BA, Gratton C, Marek S, et al. A set of functionally-defined brain regions with improved representation of the subcortex and cerebellum. *Neuroimage.* 2020;206:116290.
