# Supplementary figures and images for: Abnormal dynamic functional connectivity and topological properties of cerebellar network in male obstructive sleep apnea
Source: CNS Neurosci Ther. 2024 Jun 3;30(6):e14786. doi: 10.1111/cns.14786 (PMC11145370; doi:10.1111/cns.14786)

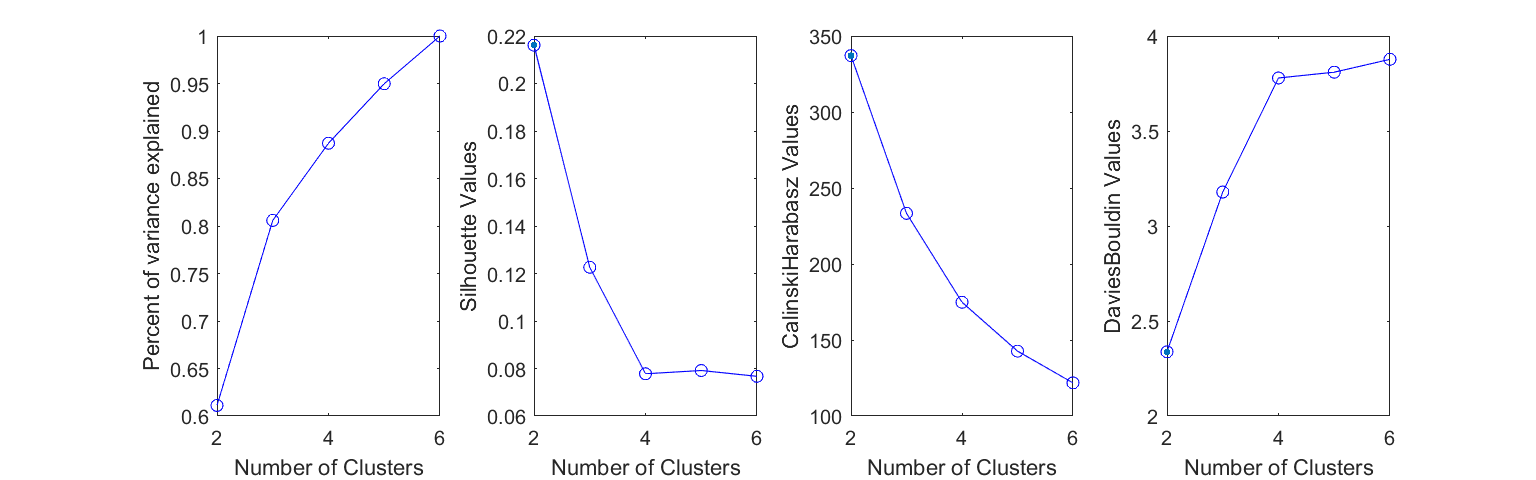

Supplement: Supplementary file 3 — Figure S2. [file CNS-30-e14786-s010.tif]

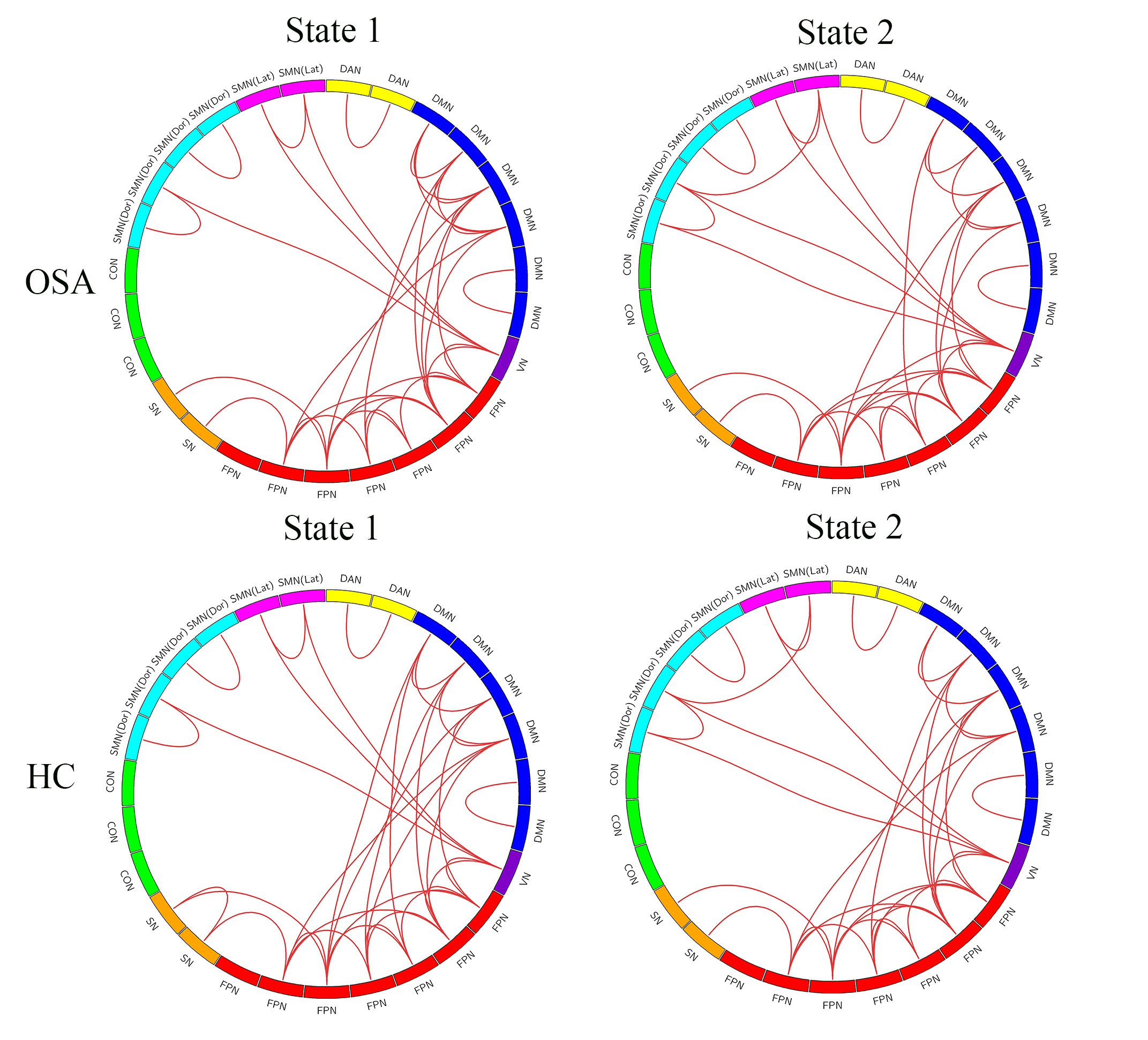

Supplement: Supplementary file 4 — Figure S3. [file CNS-30-e14786-s001.tif]

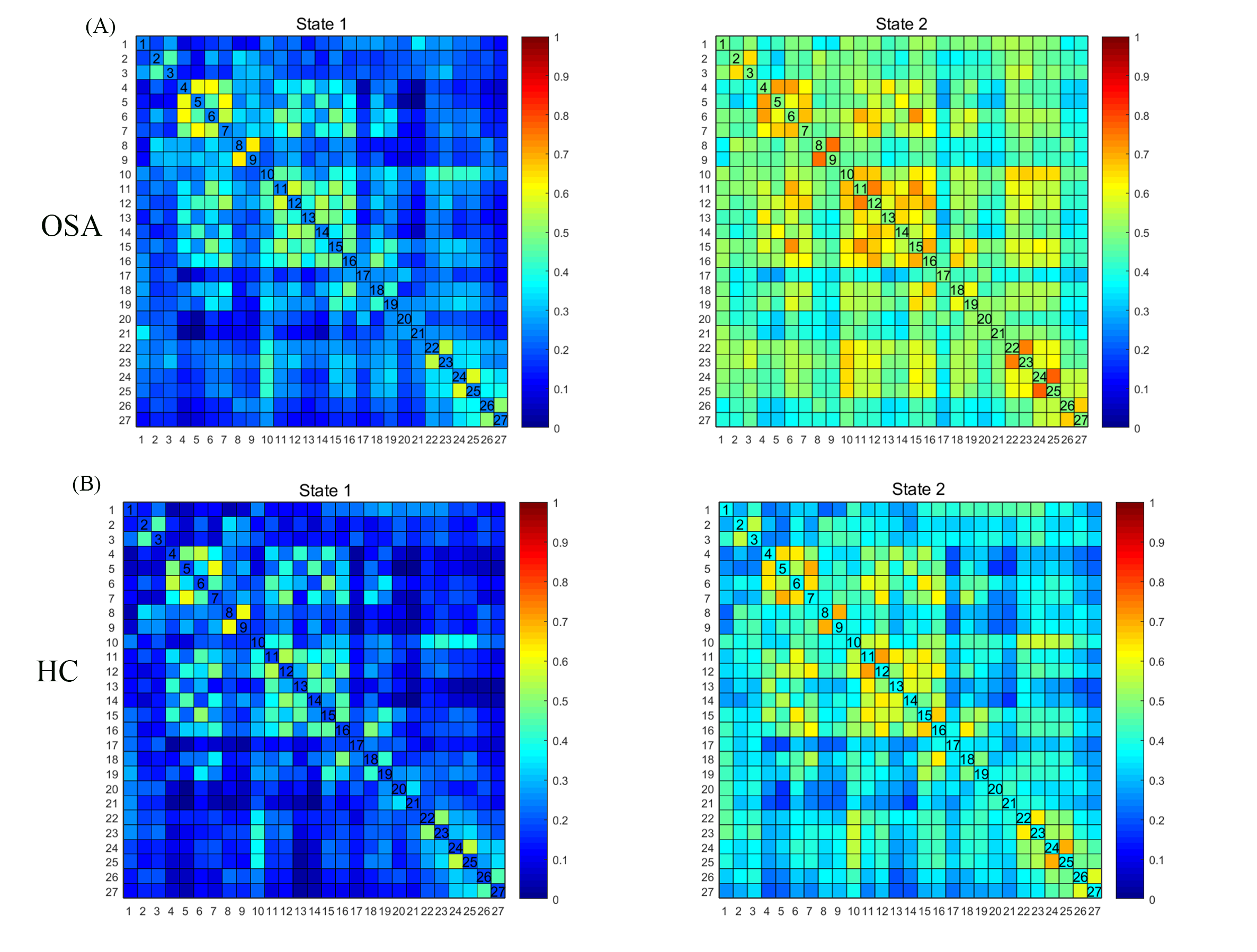

Supplement: Supplementary file 5 — Figure S4. [file CNS-30-e14786-s009.tif]

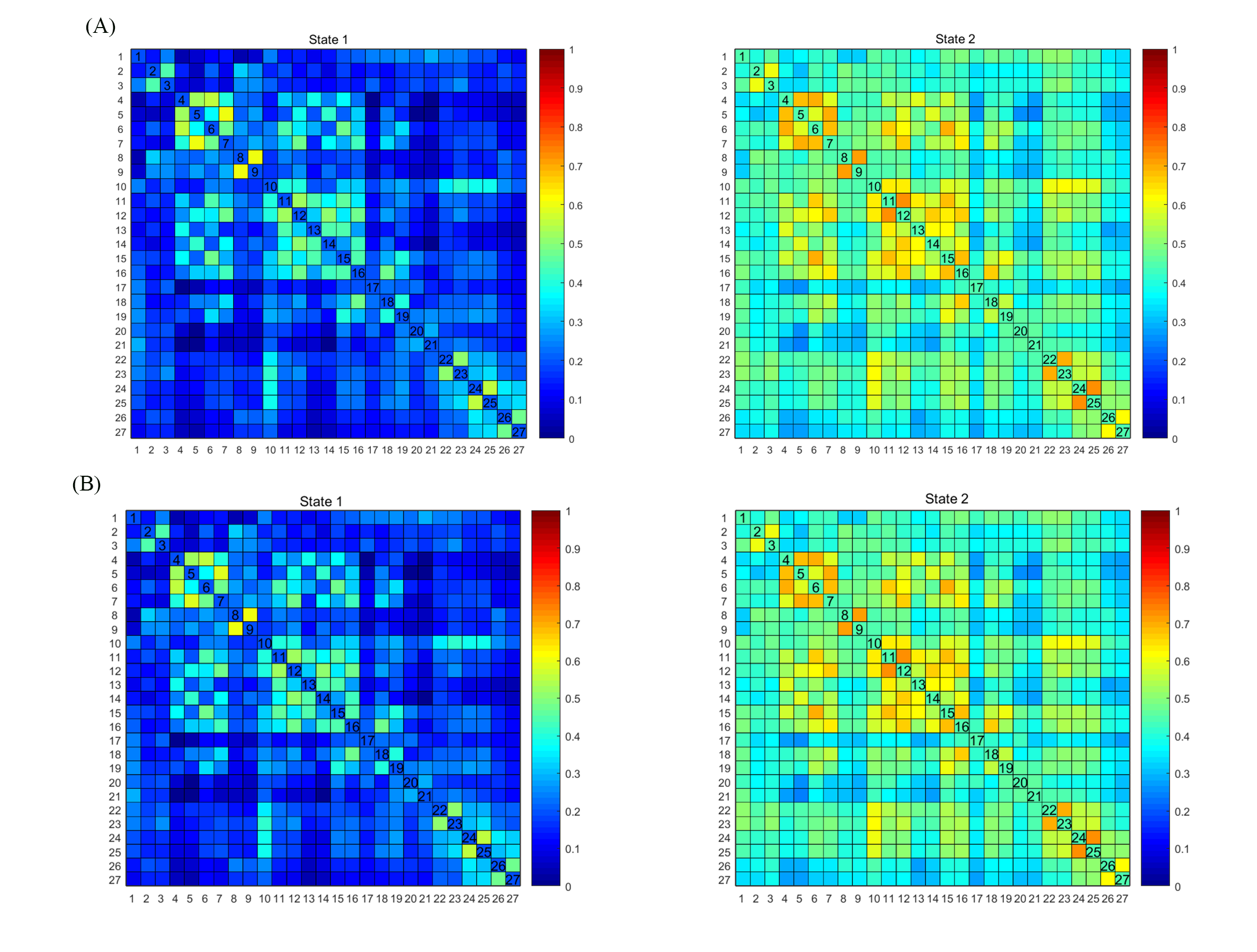

Supplement: Supplementary file 6 — Figure S5. [file CNS-30-e14786-s005.tif]

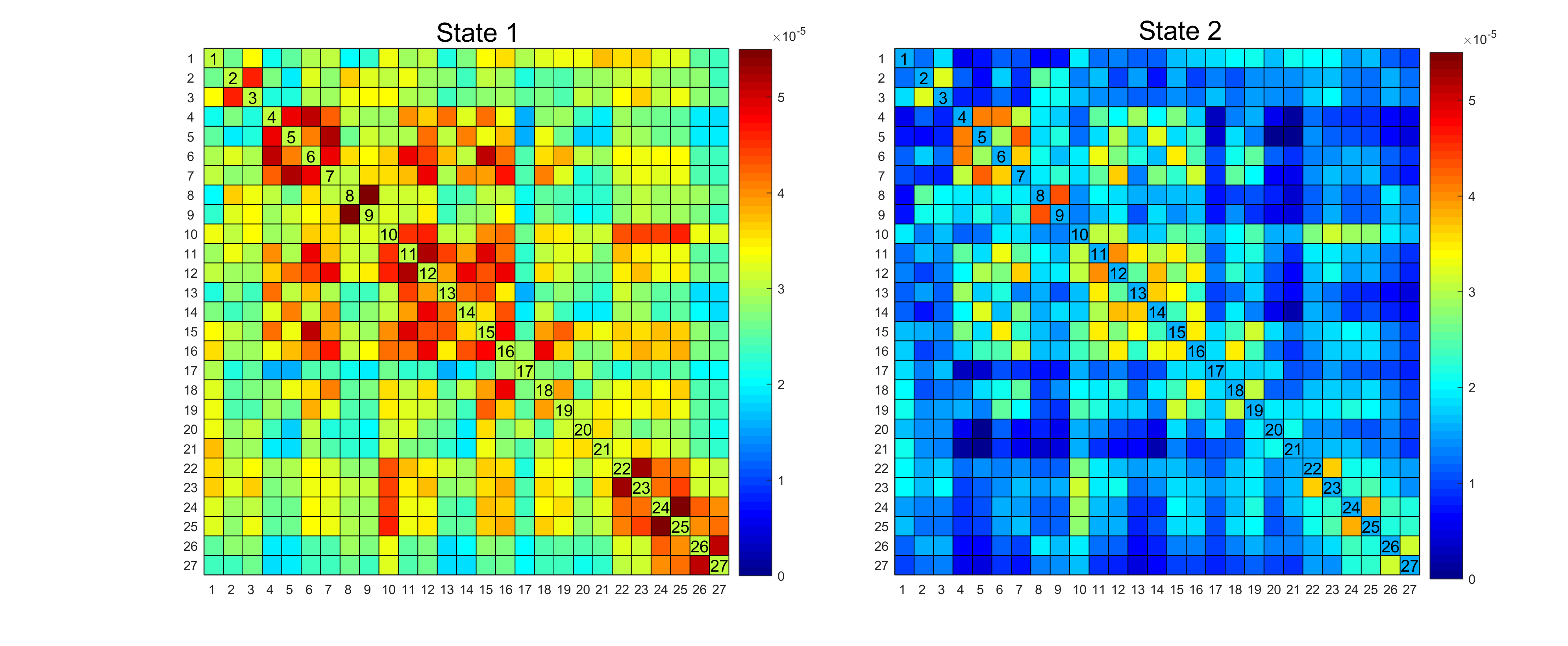

Supplement: Supplementary file 7 — Figure S6. [file CNS-30-e14786-s007.tif]

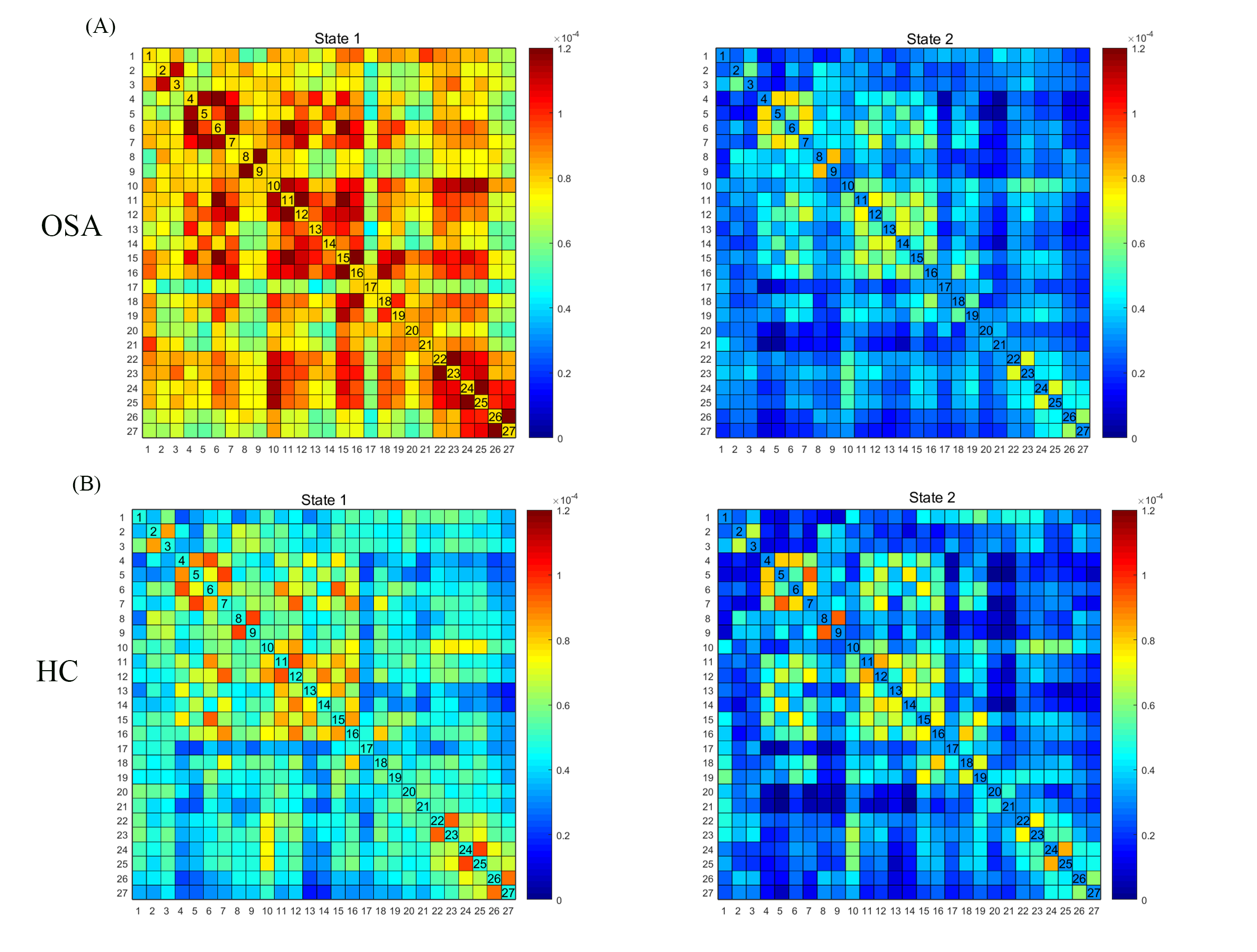

Supplement: Supplementary file 10 — Figure S9. [file CNS-30-e14786-s003.tif]
